# Pharmacodynamic modeling of synergistic birinapant/paclitaxel interactions in pancreatic cancer cells

**DOI:** 10.1186/s12885-020-07398-9

**Published:** 2020-10-23

**Authors:** Jin Niu, Xue Wang, Jun Qu, Donald E. Mager, Robert M. Straubinger

**Affiliations:** 1 Department of Pharmaceutical Sciences, University at Buffalo, State University of New York, Buffalo, New York USA; 2 Department of Cell Stress Biology, Roswell Park Cancer Institute, Buffalo, New York USA; 3 New York State Center of Excellence in Bioinformatics and Life Sciences, Buffalo, New York USA; 4 Department of Pharmacology and Therapeutics, Roswell Park Cancer Institute, Buffalo, New York 14214 USA

**Keywords:** Pharmacodynamic interaction, Synergy, Mathematical modeling, Birinapant, Paclitaxel

## Abstract

**Background:**

For most patients, pancreatic adenocarcinoma responds poorly to treatment, and novel therapeutic approaches are needed. Standard-of-care paclitaxel (PTX), combined with birinapant (BRP), a bivalent mimetic of the apoptosis antagonist SMAC (second mitochondria-derived activator of caspases), exerts synergistic killing of PANC-1 human pancreatic adenocarcinoma cells.

**Methods:**

To investigate potential mechanisms underlying this synergistic pharmacodynamic interaction, data capturing PANC-1 cell growth, apoptosis kinetics, and cell cycle distribution were integrated with high-quality IonStar-generated proteomic data capturing changes in the relative abundance of more than 3300 proteins as the cells responded to the two drugs, alone and combined.

**Results:**

PTX alone (15 nM) elicited dose-dependent G2/M-phase arrest and cellular polyploidy. Combined BRP/PTX (150/15 nM) reduced G2/M by 35% and polyploid cells by 45%, and increased apoptosis by 20%. Whereas BRP or PTX alone produced no change in the pro-apoptotic protein pJNK, and a slight increase in the anti-apoptotic protein Bcl2, the drug combination increased pJNK and decreased Bcl2 significantly compared to the vehicle control. A multi-scale, mechanism-based mathematical model was developed to investigate integrated birinapant/paclitaxel effects on temporal profiles of key proteins involved in kinetics of cell growth, death, and cell cycle distribution.

**Conclusions:**

The model, consistent with the observed reduction in the Bcl2/BAX ratio, suggests that BRP-induced apoptosis of mitotically-arrested cells is a major contributor to the synergy between BRP and PTX. Coupling proteomic and cellular response profiles with multi-scale pharmacodynamic modeling provides a quantitative mechanistic framework for evaluating pharmacodynamically-based drug-drug interactions in combination chemotherapy, and could potentially guide the development of promising drug regimens.

## Background

Eighty percent of pancreatic adenocarcinoma (PDAC) patients are not surgery candidates at diagnosis, leaving radiation and chemotherapy as their only treatment options [1]. First-line standards-of-care include multi-drug combination regimens such as FOLFIRINOX (fluorouracil, leucovorin, irinotecan, and oxaliplatin), which has been limited by its toxicity to patients of high performance status, and gemcitabine (GEM) plus nanoparticle-bound paclitaxel (Abraxane®, ABX; nab-paclitaxel) [2, 3]. Given the limited efficacy of standard therapy, identification of new drug targets and combinations is urgently needed to provide better therapeutic outcomes for PDAC. Drug combination therapies ideally should eliminate tumor cells and overcome single-agent drug resistance by combining compounds having complementary mechanisms of action. The NCI ALMANAC (A Large Matrix of Anti-Neoplastic Agent Combinations) project screened over 5000 pairs of 104 FDA-approved oncology drugs against the NCI-60 panel of human tumor cell lines to identify new, synergistic combinations [4]. However, PDAC is not represented in the NCI-60 panel [5], and there is a paucity of data for PDAC, which typically harbors large numbers of mutations in numerous core signaling pathways [6–8]. Smaller-scale screening studies have shown that few single agents or combinations show activity in PDAC [9]. Transcriptomic signatures of patient-derived organoids suggest potential biomarkers to predict chemosensitivity to FOLFIRINOX or GEM/ABX combinations [10]. However, this approach has yet to identify novel, clinically deployed combinations.

To address the need for positive modulators of standard-of-care therapy, we employed comprehensive label-free proteomic analysis of cell-level chemotherapy responses, combined with quantitative pharmacological systems analysis, in order to investigate drug interaction mechanisms that might underlie synergistic drug interactions in PDAC. Paclitaxel (PTX) is a broad-spectrum oncology drug, and a phase III study showed that PTX (as ABX) combined with GEM increased both overall- and progression-free survival by an average of 27% (1.8 mos) in metastatic PDAC patients compared to GEM alone [2]. PTX disrupts spindle dynamics during mitosis, thus inducing cell cycle delay or arrest, activation of the spindle assembly checkpoint, and accumulation of cyclin B1 [11]. The arrested cells undergo intrinsic apoptosis or mitotic slippage, depending on the balance of cellular pro- and anti-apoptotic signals [11], such as mitochondrial membrane permeability and expression of inhibitor of apoptosis (IAP) proteins such as survivin [12, 13]. Cells that pass through mitotic slippage without division may undergo further DNA replication and become polyploid, which has been associated with drug resistance [14–16]. Therefore, combination therapies that synergize with PTX to reduce the abundance of chemoresistant cells could provide therapeutic benefits in PDAC.

Birinapant (BRP) is a bivalent mimetic of the endogenous IAP antagonist SMAC (second mitochondria-derived activator of caspases) that binds to cellular inhibitor of apoptosis (cIAP) proteins 1/2 and leads to their degradation through the ubiquitin-proteasome pathway [17, 18]. The cIAPs are incorporated into TNF receptor complexes to promote pro-survival signals through NF-κB [19, 20]. Conversely, degradation of cIAPs by birinapant leads to extrinsic, TNF-mediated apoptosis [21]. These findings suggest that combination of BRP with PTX might synergize because they induce apoptosis by distinct yet convergent mechanisms. Indeed, combined BRP/PTX reduces PDAC cell proliferation synergistically in vitro [22], and proteome-level responses suggested qualitatively that metabolic-, cell cycle-, and apoptosis pathways could be involved in enhanced cell killing by this combination [22]. However, a quantitative regulatory framework linking intracellular protein expression changes to the kinetics of apoptosis and cell proliferation is lacking. Here, the pharmacodynamic interactions of BRP/PTX were investigated in the Kras ^mutant^ PANC-1 cell line by employing mathematical modeling to integrate temporal, proteome-level drug responses quantitatively with treatment-mediated transitions in intracellular signaling networks and regulation of the cell cycle and apoptosis. A novel approach, involving cluster analysis to identify temporal patterns of protein-level drug responses, was investigated as a means of identifying and modeling different drug response patterns. The clustering approach grouped proteins having similar temporal expression patterns, and was used to identify key proteins to represent the overall kinetics of protein families governing similar biological functions. This quantitative analysis of multiscale data provides new insights into the mechanisms of birinapant/paclitaxel interaction, identifies key protein-level response pathways that potentially underlie their synergistic interactions, and provides quantitative estimates of the contribution of specific protein expression changes to cell cycle progression.

## Methods

### Reagents

Paclitaxel, sulforhodamine B sodium salt (SRB), trichloroacetic acid, dimethylsulfoxide (DMSO), and Tris were from Sigma-Aldrich (St. Louis, MO). Propidium iodide (PI)/RNase Staining Buffer and the AnnexinV-phycoerythrin (PE)/7-aminoactinomycin D (7-AAD) Apoptosis Detection Kit were from BD Pharmingen (San Diego, CA). The ACCUTASE cell detachment solution was from EMD Millipore (Temecula, CA). Birinapant was a gift of TetraLogic Pharmaceuticals (Malvern, PA). Stock solutions of PTX (10 mM) and BRP (30 mM) were prepared in DMSO and stored at − 20 °C until use. When diluted to final concentrations in cell culture medium, the DMSO concentration was below 0.1% (v/v) and did not perturb cell growth.

### Cell culture

The human pancreatic cancer cell line PANC-1 was obtained from the American Type Culture Collection (Rockville, MD). It harbors several common mutations in pancreatic cancer, including KRAS^G12D^, TP53^R273H^, and homozygous deletion of CDKN2A [23]. Cells were cultured in Dulbecco’s Modified Eagle’s Medium (Cellgro, Manassas, VA) containing 10% (v/v) fetal bovine serum (Atlanta Biological, Lawrenceville, VA) in a humidified atmosphere with 5% CO_2_ at 37 °C. Cells were passaged at 80 ~ 90% confluence using 0.05% trypsin with 0.53 mM EDTA (Gibco BRL, Gaithersburg, MD).

### Cell proliferation assay

Cells were seeded in 96-well plates at a density of 3.0 × 10^3^ cells/well, allowed to adhere overnight, and at T0 (approx. 18 h later), they were treated with varied concentrations of PTX (2.5–60 nM) and/or BRP (15–1000 nM). The vehicle control was treated with 0.1% (v/v) DMSO. At intervals after initiation of treatment (24, 48, 72, 96, 120 h), cell proliferation was quantified by SRB assay [24], which exhibited good linearity over the optical density range of 0.036–2.22 (R^2^ = 0.97). Cell proliferation was normalized to the mean cell density at the initiation of treatment (T0). Because the SRB assay quantifies total cellular protein, rather than cell number, it may overestimate cell number when drug treatment conditions generate a significant proportion of polyploid cells. To estimate the effect of polyploid cells on cell count, we used the cell cycle model (below) to simulate the number of diploid and polyploid cells in each treatment group, based on experimental data for the fraction of polyploid cells. Assuming that the protein content of a polyploid cell is twice that of diploid cells (given the short duration of the experiment), the “apparent” cell number is calculated as *N*_*diploid*_ + 2 · *N*_*polyploid*_. A correction factor (CF) was then calculated as:

$$ CF=\frac{true\ number}{apparant\ number}=\frac{N_{diploid}+{N}_{polyploid}}{N_{diploid}+2\cdotp {N}_{polyploid}} $$

and was applied to the measured optical densities for each experimental group. The range of the CF was 0.70–1.0 for PTX-treated group and 0.73–1.0 for the BRP/PTX combination group. The effect of this correction to cell number is described in *Results*.

### Cell cycle analysis

Cell cycle distribution was analyzed as previously described [25]. Cells were seeded in 6-well plates (2.0 × 10^5^ cells/well), allowed to adhere overnight, and at T0 were exposed to PTX (5–50 nM) and/or BRP (0.1–3 μM). The vehicle control was treated with 0.1% (v/v) DMSO. Just before drug exposure at T0, and after 17, 48, and 72 h of drug exposure, adherent cells were harvested, counted by Coulter counter (Hialeah, FL) and stained with PI/RNase staining buffer. At least 10,000 events from triplicate samples were collected using a FACSCalibur flow cytometer (Becton–Dickinson, Mansfield, MA), and cell cycle distributions were analyzed using ModFit 3.2 (Verity Software House, Topsham, ME). The fitted ModFit curves were smooth, and debris and aggregates were < 5%. Cells in the G0/G1- and G2/M-phases were identified based upon DNA content of 2 N and 4 N, and the intensity ratio for G2/G1 was 1.8–1.9. DNA content for cells in S-phase was between 2 N to 4 N; content > 4 N was defined as polyploid. The sub-G1 population was minimal and excluded from quantification.

### AnnexinV/7-AAD apoptosis assay

The apoptotic cell fraction was quantified using AnnexinV/7-AAD staining and flow cytometry as previously described [25]. PANC-1 cells were seeded as above and exposed to BRP (0.1–3 μM) and/or PTX (15–50 nM), with 0.1% DMSO-treated controls. At intervals, cells were harvested as described above for flow cytometry. Data were analyzed using FCS Express5 Flow Cytometry software (DeNovo Software, Los Angeles, CA). Four cell populations were identified based on their fluorescence staining: (i) live (AnnexinV^−^/7-AAD^−^), (ii) early apoptotic (AnnexinV^+^/7-AAD^−^), (iii) necrotic/late apoptotic (AnnexinV^+^/7-AAD^+^), and (iv) ‘other’ (AnnexinV^−^/7-AAD^+^) which was < 1% for all samples. Apoptotic cells were quantified as the total AnnexinV^+^ population.

### Large-scale temporal proteomic expression analysis

Quantitative proteomic data were obtained at intervals over 72 h of exposure to BRP and PTX, alone and combined, using the label-free IonStar workflow, as described previously [26], and 4069 proteins were quantified according to stringent criteria: ≥2 quantified peptides per quantified protein; False Discovery Rate for peptide identification ≤0.1%, for protein identification ≤1%, with *p* < 0.05 [22]. Unperturbed baseline temporal expression profiles of 4110 proteins were quantified in control cells using the same workflow [27]. A total of 3325 proteins were quantified in all samples in two experiments. A quantile normalization method was applied to correct for between-batch differences, and provided relative change in expression for treatment vs. control [28]. Normalization is essential for comparative proteomic analysis, and numerous approaches to normalization exist, including normalization of each treatment group to its own ‘time zero’ point, or normalization of treated vs. vehicle control samples at each time point. All normalization approaches possess strengths and weaknesses. Here, the latter approach was employed, and data were then transformed to log2 scale. This normalization approach was chosen because it reveals the effect of drug intervention as cells proliferate in culture. A disadvantage is that unperturbed control cells eventually transition from exponential growth to contact inhibition when approaching confluency, and sub-confluent, drug-treated cells may not experience confluency effects on cell cycle progression. Previously we evaluated the effect of normalization upon the quantitative conclusions drawn from data analysis by modeling [29], and observed that the normalization strategy did not affect the robustness of estimation of turnover and drug effect parameters, nor their interpretation.

Temporal proteomic profiles were clustered into 9 time-series using a k-means clustering method (Short Time-series Expression Miner software, STEM [30];), under the assumption that proteins collaborating mechanistically in different phases of drug response would share similar temporal expression patterns [31]. The temporal profile of each cluster is represented as mean values over time. Protein expression was validated orthogonally using Western blots that were quantified using ImageJ; bands were normalized to GAPDH or β-actin expression [22]. Analysis of protein expression changes based upon Gene Ontology (GO) annotation for biological process, cellular component, and molecular function was performed using the DAVID (Database for Annotation, Visualization and Integrated Discovery) Bioinformatics Resource v6.7 (*https://david.ncifcrf.gov/*) as described previously [22].

### Mathematical modeling

#### Cell growth kinetic model

**Fig. 1:**
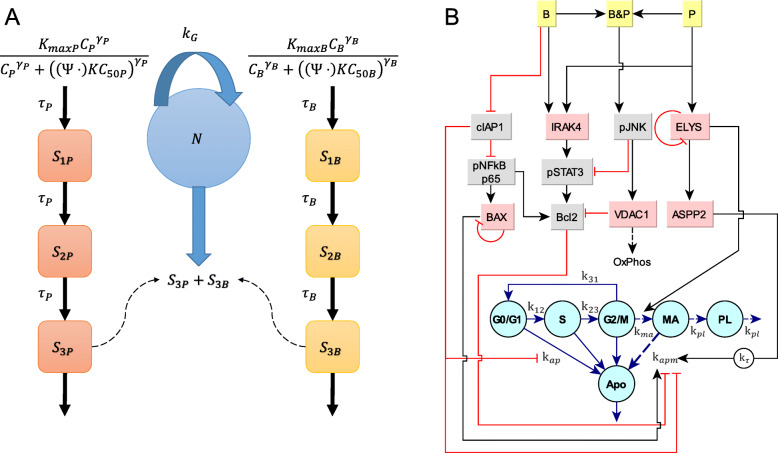
Schematics of cell proliferation model and proteomics-based cell cycle and apoptosis model of PANC-1 cells exposed to paclitaxel and birinapant. **a** Model structure for PANC-1 cell growth inhibition by paclitaxel and birinapant, alone and combined. B: birinapant; P: paclitaxel. The cell number N (blue circle) increases as cells proliferate in an exponential manner, with net growth rate constant k_G_. Concentration-dependent cytotoxic signals for the two drugs are modeled by nonlinear Hill functions with transduction delays (rounded rectangles), and mediate removal of cells (cell killing; downward blue arrow) from the population. The killing signals are additive. The Ψ drug interaction term is fixed to 1 for single-drug treatment but is fitted for drug combinations. **b** Structure of the proteomics-based cell cycle and apoptosis model for cells exposed to birinapant (B) and paclitaxel (P). The BRP/PTX combination is represent as B&P. Pink boxes: proteins quantified by proteomics; grey boxes: proteins measured by western blot; circles: cells in different cell cycle stages or undergoing apoptosis. Activation of a protein/signal is denoted by a black arrow, inhibition by a red bar. Each live cell progresses through G_0_/G_1_, S, and G_2_/M phases and divides into two progeny cells. Live cells can also undergo spontaneous apoptosis (Apo). Birinapant acts by accelerating degradation of cIAP1, an inhibitor of apoptosis. Paclitaxel-induced mitotic arrest (MA) is mediated by ELYS, and the mitotically-arrested cells are prone to apoptosis, regulated by cIAP1, BAX, Bcl2, and the delayed signal of ASPP2. The mitotically-arrested cells may also undergo mitotic slippage and become polyploid cells (PL). The transition rate constants between the cell cycle stages and to apoptosis are represented by the ‘k’ parameters, described in Table 1

A pharmacodynamic model that included an exponential cell growth function with concentration-dependent cytotoxicity was developed to quantify the nature of interaction between PTX and BRP on PANC-1 cell growth kinetics, (Fig. 1a). The unperturbed growth of PANC-1 cells in the control group is represented by the first-order growth rate constant k_G_. Nonlinear Hill functions with time-dependent signal transduction delays were used to describe the temporal effects of the two drugs [32–34]. A signal distribution model was parameterized with the maximum killing rate constant (K_max_) for each drug, the concentration to achieve 50% of the maximum killing (KC_50_), and a mean transit time τ for the signal transduction delay. An interaction term Ψ was included to characterize the nature of the drug-drug interaction: for Ψ < 1, the combination is synergistic (supra-additive); for Ψ = 1, it is additive; for Ψ > 1, it is antagonistic (sub-additive). Zero to five transduction delay compartments were tested during model development, and model selection was based on the lowest Akaike information criterion (AIC) value and visual examination of the weighted residuals (WRES) distribution. The variance model was constructed as Var = (δ + σ · Y(t))^2^, with Y(t) as the model-predicted value, and *δ* and *σ* as estimated variance model parameters. ADAPT5 [35] was used for model fitting, using the maximum likelihood estimation method. Supplementary Table S1 shows the complete set of equations.

#### Cell cycle and apoptosis model based on large-scale proteomics analysis

A multi-scale, mathematical network model was developed using a sequential model-fitting strategy to integrate quantitatively pharmacodynamic endpoints such as the temporal changes in cell cycle progression, expression of drug-responsive proteins, and apoptosis of cells during exposure to BRP/PTX (Fig. 1b). First, a model for protein interactions was constructed (Fig. 1b, boxes) based on literature describing relevant proteins that contribute to the mechanisms of action of PTX and BRP, and their known interactions (Supplementary Table S2). For example, cIAP1 was included in the model as it is a known direct target of BRP [18]. Temporal clustering of the proteomic expression profiles showed that mitotic spindle and kinetochore proteins exhibited similar temporal profiles after PTX treatment. Therefore, one representative protein, the kinetochore/nuclear pore complex protein ELYS, was selected to represent the proteins in this functional group that are proximal targets of PTX. NF-κB was included because it is a key protein involved in BRP-induced apoptosis signaling pathways mediated by cIAP in pancreatic cancer [25]. Additional proteins were included if their expression in the BRP/PTX combination group differed from their expression in either single-agent treatment group. For example, mitochondrial protein VDAC1 (voltage- dependent anion channel 1) was not perturbed by PTX or BRP alone, but was increased by the combination of BRP/PTX. The dynamics of individual proteins were then described using an indirect response model [36], with a first-order degradation rate constant k_deg_ for the turnover of each protein. The baseline or initial value for any protein is 1 in the absence of treatment, and thus the synthesis rate constant (k_syn_) is a fixed function defined as:

$$ {\mathrm{k}}_{\mathrm{syn}}={\mathrm{k}}_{\mathrm{deg}}\times \mathrm{baseline}={\mathrm{k}}_{\mathrm{deg}} $$

In the model, BRP leads to cIAP1 degradation and subsequently induces phosphorylation of the p65 NF-κB subunit, which regulates the transcription of both pro-apoptotic BAX and anti-apoptotic Bcl2. All down- or up-regulated processes were represented with a factor multiplied by the protein’s synthesis rate as: k_syn_ × (1 − *Inh*_B_) or k_syn_ × (1 + *Sti*_P_), where *Inh* or *Sti* represent inhibition or stimulation, and B and P indicate BRP and PTX (Supplementary Table S3, Eqs. 4–12). BAX regulates the expression of itself through feedback mechanisms. In the model, PTX induces accumulation of ELYS, a kinetochore and nuclear pore complex protein, and subsequently activates ASPP2 (apoptosis-stimulating protein of p53), which can activate intrinsic apoptosis after it binds to p53 family proteins. Both BRP and PTX are assumed to induce IRAK4 expression, which leads to STAT3 phosphorylation and activation. Phosphorylated STAT3 then induces Bcl2 expression. Only when the two drugs are combined, the pro-apoptotic signal protein JNK is phosphorylated and induces the expression of VDAC1. VDAC1 then inhibits Bcl2 expression, and its expression represents mitochondrial activities such as oxidative phosphorylation.

This structural model was used to fit the large-scale temporal protein expression data from PANC-1 cells exposed to BRP (100 nM) and/or PTX (10 nM) for up to 72 h. A constant error model (*Var* = *δ*_*p*_) was used for all fold-changes in log scale. Model fitting was conducted sequentially with Matlab 2018a, using the nonlinear regression function “*nlinfit*”. Parameters associated with upstream relationships were estimated first, and then fixed for modeling downstream processes. For example, parameters for BRP effects on its direct targets (e.g., cIAP) were estimated and then fixed to the estimated value for modeling the downstream interaction between cIAP and NF-κB.

An additional component of the cell cycle and apoptosis model (Fig. 1b) was developed to capture the kinetics of cell cycle progression and estimate the rate constants controlling cell cycle progression, density-dependent inhibition, and naturally-occurring apoptosis in untreated controls. In the absence of treatment, proliferating cells progress through G_0_/G_1_, S, and G_2_/M phases, and then cytokinesis, according to the first-order transition rate constants k_12_, k_23_, and k_31_. The values of these rate constants are established by co-modeling all cell cycle data, and govern the mean transit time through each phase. In the model, cells can undergo spontaneous apoptosis according to a first-order rate constant, k_ap_, and the apoptotic cell pool (Apo) undergoes turnover according to k_ap_. As cell density increases, nutrients and growth factors are consumed, which cause cells to exit the cell cycle and accumulate in G_0_ phase [37]. Therefore, cell density-dependent inhibition (I_0_), which slows the G_0_/G_1_ to S phase progression gradually, is modeled with a Gompertz differential equation [38], in which the capacity parameter *N*_*max*_ represents the maximum number of cycling cells under the culture conditions. The parameters of this model were estimated by fitting it to unperturbed vehicle control data for PANC-1 cell cycle progression, apoptosis, and cell proliferation over 72 h. A constant error model was used for cell cycle- and apoptosis percentages, and the variance model for total cell count was *Var* = (*δ*_*n*_ + *σ*_*n*_ × *Y*(*t*))^2^. Model fitting was conducted with ADAPT5 using the maximum likelihood estimation.

The temporal protein interaction network and cell cycle/apoptosis models were ultimately linked. Because cIAP1 is a natural inhibitor of apoptosis, the abundance of cIAP1 modifies the natural progression of apoptosis and is represented as ${\mathrm{k}}_{ap}\times {cIAP}^{\gamma_{cIAP}}$, where γ_cIAP_ < 0 indicates inhibition. The kinetochore forms during mitosis and the response of the protein ELYS is used to represent the group of kinetochore proteins that stimulates the unperturbed cells in G2/M phase to transition into a mitotically-arrested (*M*_*A*_) stage when ELYS expression is upregulated from baseline. This stimulation is represented as ${k}_{ma}={k}_{ma0}\times {(ELYS)}^{\gamma_{ELYS}}\times \left( ELYS>1\right)$, where *k*_*ma*_ is the rate constant controlling the transition from G2/M phase to *M*_*A*_. When ELYS expression is less than or equal to the baseline, *k*_*ma*_ = 0 and cells progress without mitotic arrest. Mitotically-arrested cells either undergo apoptosis with a rate constant of k_*apm*0_, which is modified by BAX, Bcl2, cIAP1 and ASPP2 expression, or they escape apoptosis through mitotic slippage and form polyploid cells according to a first-order rate constant k_*pl*_. The models of both direct- or delayed signals were tested for proteins that stimulate k_*apm*0_ in order to capture the temporal dynamics of the apoptotic cell fraction. Incorporating a delayed signal for ASPP2 induction improved model fitting based on the lowest mean squared error and visual inspection of observed and predicted values. Polyploid cells were assumed to be apoptosis-resistant and were quantified experimentally as having a cellular DNA content of > 4 N. The sum of G2/M and mitotically-arrested cell populations was quantified as tetraploid cells (DNA content =4 N). Initially, drug effects on cell cycle and apoptosis were constructed to link drug concentrations directly to the rate constants for mitotic arrest and apoptosis induction. All other parameters for cell cycle progression were fixed to the estimated values obtained from the control group. These concentration-dependent relationships were subsequently replaced with power coefficients on corresponding proteins to stimulate apoptosis or mitotic arrest (e.g., *γ*_*cIAP*_ and *γ*_*ELYS*_), and parameters from the protein interaction network were fixed to estimated values. Model fitting was conducted with Matlab 2018a, using “*nlinfit*” function. All model equations are listed in Supplementary Table S3.

#### Major assumptions in the models

Several major assumptions were employed in the development of the models. One is that both drugs are stable in the cell culture medium for at least 72 h. Paclitaxel was reported to undergo < 10% hydrolysis in cell culture medium over 96 h [39]. The stability of birinapant in cell culture media was not available in the literature, but can be inferred from the expression of cIAP1 (see *Results*). A second assumption is that the drug-mediated changes in protein expression are concentration-dependent, and that protein interactions, and cell cycle and apoptosis transitions, are dependent on the fold-change in expression of the relevant protein. A third assumption is that it was appropriate to represent protein expression as fold-change relative to control, rather than as an absolute concentration. Thus the turnover parameter k_deg_ is for protein fold-change, as opposed to representing the turnover of the actual protein molecules. Fourth, additive and independent mechanism(s) of action were assumed for the two drugs. In the cell growth kinetic model (Fig. 1a), for example, the cytotoxic signals of BRP and PTX are added to each other, with an additional interaction term *ψ* to represent the effect of one drug on the *KC*_50_ value of the other. In the proteomics-based cell cycle and apoptosis model (Fig. 1b), it was assumed that the points of interaction between BRP and PTX were revealed by those proteins whose expression profiles in the combination group could not be accounted for by the single-agent treatment responses. Finally, biological processes in the model, such as the transition of cells through the cell cycle phases, were described with first-order reactions for both the entry and exit reactions of the process (cycle phase). That is, the total quantity exiting would be the first-order rate constant × the quantity of the species, and the quantity transitioning into that phase would be calculated in similar manner. For example, cells exit M phase at the rate of *k*_31_ × *M*, and at the same time, the number of cells that reenter the subsequent G0/G1 phase following cell division is described by a rate of 2 × *k*_31_ × *M*.

## Results

### Effects of paclitaxel and birinapant on PANC-1 cell proliferation

**Fig. 2:**
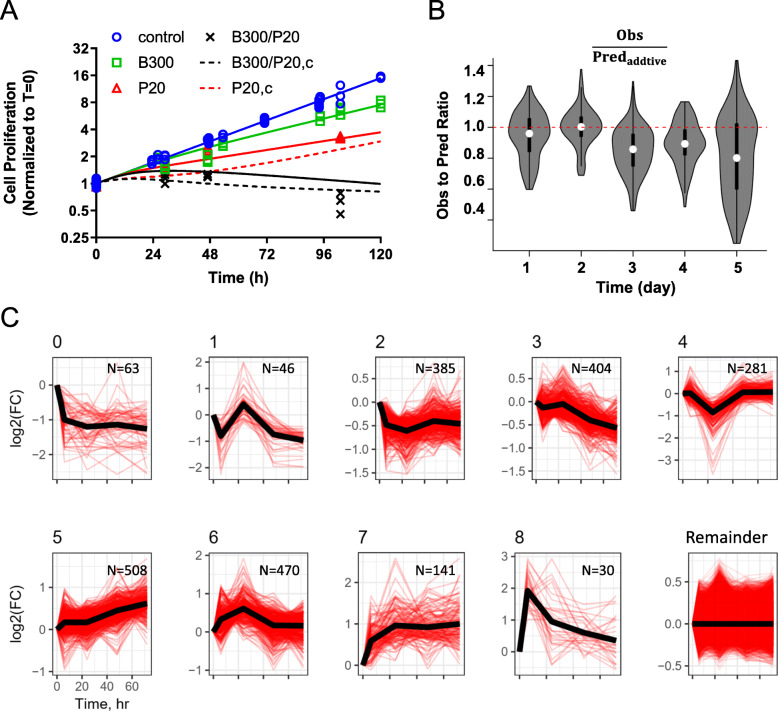
PANC-1 cell growth and clustering of proteomic expression during exposure to paclitaxel and birinapant. **a** Representative model-fitted profiles (solid lines) of PANC-1 cell proliferation measured experimentally (symbols) over 120 h of exposure to vehicle control (blue circles), single-agent BRP (green squares), PTX (red triangles), and BRP/PTX combined (black crosses). Observed cell proliferation is normalized to t = 0 h, and all observations are in triplicate. Dashed lines are cell number after correction for the polyploid cells. **b** The ratio of observed data to values computed assuming additive drug interaction for the BRP/PTX combinations. White circles represent median values, vertical black bars represent the 2nd to 3rd quartiles, the grey shapes of violins represent histograms of the data, and horizontal bars at the ends of violins show data ranges. **c**. The clustering of temporal protein expression responses to birinapant/paclitaxel treatment. The k-means clustering algorithm was applied to quantitative proteomics data for PANC-1 cells exposed to vehicle (0.1% DMSO), or 100 nM birinapant and 10 nM paclitaxel, alone or combined, for 6, 24, 48 and 72 h

Initial proteome-level analysis revealed the impact of combined PTX and BRP on a wide range of vital cellular functions that could inhibit cell proliferation and promote cell death [22]. In order to identify key points of drug interaction that underlie the observed drug synergy on pancreatic cancer cells, we developed quantitative pharmacodynamic and network interaction models as an approach to extract a more mechanistic understanding of the synergy from temporal changes in large-scale proteomic data. PANC-1 cell growth kinetics were quantified over 5 days of exposure to a range of 6 PTX concentrations, 4 BRP concentrations, and 24 paired BRP/PTX combinations that encompass the concentration ranges over which synergistic drug interactions were observed. A mathematical pharmacodynamic model (Fig. 1a) that assumes exponential cell growth and delayed, concentration-dependent cytotoxicity was fitted to the data. A comparison of representative observed- and model-fitted growth kinetics is shown in Fig. 2a, with expanded results and estimated parameters shown in Supplementary Fig. S1 and Table S4. The net growth rate constant for PANC-1 cells was estimated as 0.0225 h^− 1^, corresponding to a doubling time of 30.8 h, which is consistent with the literature [40]. PTX exerted greater cytotoxicity than did BRP, with a greater killing capacity K_max_ of 0.0233 h^− 1^ for PTX, compared to 0.0153 h^− 1^ for BRP. PTX potency was also greater (KC_50_ = 18.3 nM for PTX vs. 277 nM for BRP). Because *K*_*maxP*_ > *k*_*G*_ > *K*_*maxB*_, i.e.*,* the PTX killing rate was greater than the cell proliferation rate, PTX as a single agent could eliminate cells completely under these culture conditions, given a sufficiently high concentration and duration of exposure, whereas BRP as a single agent can only retard cell growth. When growth kinetics were analyzed with the cell growth kinetic model of Fig. 1a over concentration ranges of the two drugs**,** combined BRP/PTX exhibited synergy, with a time-independent drug interaction term Ψ of 0.69 (95% confidence interval for Ψ = 0.64–0.74). Combining PTX with BRP decreased the *KC*_50_ for each drug by 31% compared to single-drug treatment. Because Ψ =1 (additive interaction) is outside the 95% confidence interval, the probability to observe additive interaction is < 5%, which is a typical threshold for statistical hypothesis testing. The total cell numbers obtained by the SRB assay could overestimate the true cell counts for treatments with high PTX concentrations; SRB measures protein mass and not cell number, and a fraction of cells transitioned into the polyploid state. We investigated the impact of potential overestimation on cell numbers (*Methods*), and used the cell cycle analysis model to simulate corrected cell number data (Fig. 2a, dashed lines, Supplement Table S5). The simulation confirmed not only that the impact of polyploid cells on cell number was similar for the PTX and BRP/PTX treated groups, but also that the correction changed cell numbers in those groups by < 10%. Thus, estimation of the BRP-PTX interaction parameter Ψ is virtually unaffected by the emergence of polyploid cells.

Synergistic interactions were not observed with shorter (< 48 h) durations of drug exposure because of the temporal delay of the drug effect signal, and because the assay dynamic range is small at early exposure times. The model was used to estimate when drug synergy might be observed experimentally. The ratios of observed vs. predicted cell growth were calculated for all BRP/PTX combinations, assuming additive interaction (Ψ = 1). The mean of these ratios was < 1 on days 3–5 of drug exposure (Fig. 2b), indicating that synergistic effects emerged at ≥72 h.

### BRP/PTX effects at the proteome level

Temporal profiles were obtained for 3325 quantified proteins quantified in all samples in four data sets derived from two experiments investigating PANC-1 cells exposed to 100 nM BRP and 10 nM PTX, alone and combined, along with a vehicle control [22]. The expression change of each of the quantified proteins was normalized to the vehicle control at the corresponding time point. To discover underlying temporal patterns within this large dataset, these expression profiles were analyzed using STEM software [30]. A total of 9 temporal clusters was identified based on the lowest absolute value of the maximum deviation from a cluster mean (Fig. 2c, Supplementary Fig. S2). Each cluster consisted of between 30 and 508 temporal protein expression profiles, and the mean time-course of each cluster was calculated.

**Fig. 3:**
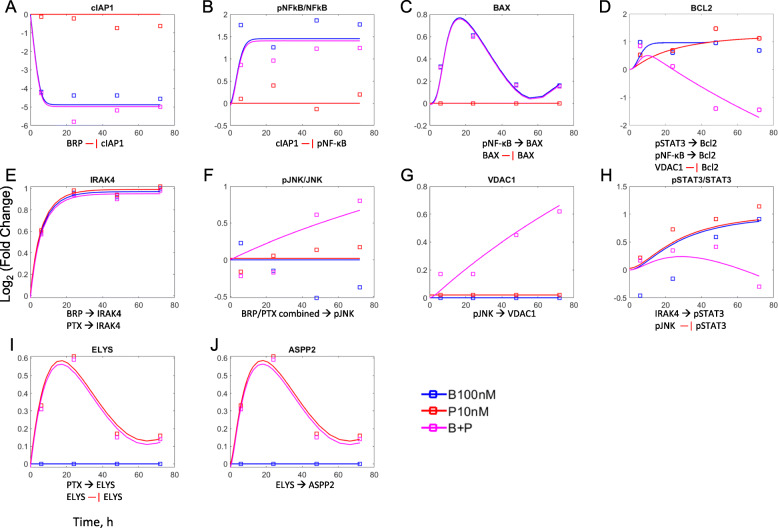
Treatment-mediated expression profiles of proteins relevant to birinapant/paclitaxel pharmacodynamic interactions. PANC-1 cells were exposed to vehicle, 10 nM PTX, 100 nM BRP, or combined BRP/PTX (100/10 nM) for up to 72 h. Treatment-mediated changes are compared to the vehicle controls for each time point. For proteins quantified using the IonStar proteomic workflow (ASPP2, BAX, ELYS, IRAK4 and VDAC1), the temporal profiles were clustered, as described in Methods, and the mean temporal cluster profiles are shown. For proteins quantified by western blot (BCL2, cIAP1, phospho-JNK, NF-κB and STAT), the expression is normalized to housekeeping proteins GAPDH or β-actin. Squares represent the treatment-mediated fold-change of a protein, whereas lines represent the fitting results from the protein interaction model. Blue: 100 nM BRP; red: 10 nM PTX; magenta: BRP/PTX (100/10 nM) combined. The protein interactions are noted at the bottom of each panel. ➔: activation; ―| inhibition

Expression data for 10 drug-responding proteins considered central to specific cell functional groups, based on DAVID analysis (https://david.ncifcrf.gov/), were extracted from relevant clusters of proteomic data or from Western Blot analysis. The cell cycle/apoptosis network model developed for protein interactions (Fig. 1b) was fitted to the expression data, and the observed and model-fitted profiles are shown in Fig. 3. The temporal responses of cIAP1 were also analyzed, owing to its apoptosis-inhibiting function and role as a direct target for BRP [18]. PTX alone had little effect upon cIAP1 abundance, but BRP or BRP/PTX led to its rapid disappearance (≤6 h; Fig. 3a). The estimated degradation rate constant was 0.649 h^− 1^ (Table 1), corresponding to a half-life of 1.07 h, which is consistent with rapid birinapant-induced degradation of cIAP1/2 [18]. The inhibition of cIAP1 by 100 nM BRP was nearly complete (*Inh*_*B*_= 97%), and it remained suppressed for 72 h of exposure. The concentration dependence of BRP-mediated supression of cIAP1 was not investigated here, but in birinapant-sensitive breast cancer cells, BRP concentrations as low as 10 nM have achieved 90% cIAP1 loss [18]. Temporal responses of other mediators of apoptosis were analyzed. NF-κB has a complex role in apoptosis, especially in apoptosis induced by BRP via cIAP degradation [18, 27]. Both BRP and BRP/PTX mediated a 2.8-fold increase (equal to 1.5 on log2 scale) in the phosphorylation of the p65 subunit of NF-κB compared to its baseline control (Fig. 3b), which is consistent with reports that BRP increased phosphorylated p65 in the absence of TNFα ligand, but decreased it below baseline in the presence of TNFα [41, 42]. NF-κB induces mitochondrial proteins BAX and Bcl2, both of which influence mitochondria-mediated apoptosis. Over the first 24 h, BRP or BRP/PTX increased pro-apoptotic BAX 1.5-fold over control (0.58 on log2 scale; Fig. 3c), followed by a return toward baseline that was likely driven by auto-feedback regulation [43]. Pro-survival protein Bcl2 increased to 1.9-fold over controls in response to BRP or PTX alone, but after 24 h of exposure, the BRP/PTX combination alone decreased its abundance to 0.4-fold of control at 48-72 h (Fig. 3d). The initial increase in Bcl2 may reflect increased transcription driven by phosphorylated p65, but the later decrease in Bcl2 likely results from other mechanism(s). Bcl2 is regulated by transcription factor STAT3 [44] and mitochondrial membrane protein VDAC1 [45], as well as by their upstream regulators IRAK4 and JNK. In this study, IRAK4 increased 2-fold over control with any drug treatment (Fig. 3e). Only the BRP/PTX combination increased phosphorylated JNK (1.7-fold; ig. 3F) and VDAC1 (1.5-fold; Fig. 3g). Whereas phosphorylated STAT3 increased by 2-fold with BRP or PTX treatment (Fig. 3h), combined BRP/PTX prevented the increase, because phosphorylated JNK, which inhibits STAT3 phosphorylation [46], was increased. Overall, the proteomic data are consistent with the main mechanism of action of BRP being promotion of cIAP degradation, which initiates NF-κB signaling. The NF-κB signal then propagates to mitochondrial proteins such as BAX and Bcl2, thereby increasing intrinsic apoptosis. The data also suggest that the BRP/PTX combination drives the balance of apoptosis regulators toward a pro-apoptotic, anti-survival status.

**Table 1:** Estimated parameters for the protein-based cell cycle and apoptosis model

	Parameter		Unit	Estimate (CV%)	Comment	Drug-specific?
Protein interactions model	${\mathrm{k}}_{\deg_{\mathrm{i}}}$	Synthesis- or degradation rate constant for the turnover of protein i. (protein-specific parameters)	h^−1^	0.649 (16.8)	Bcl2, cIAP1 and pNF-κB (pp65)	N
				0.127 (6.94)	IRAK4	N
				8.26 × 10^− 3^ (38.9)	pJNK	N
				3.16 × 10^−2^ (46.7)	pSTAT3	N
				0.696 (165)	VDAC1	N
				2.80 × 10^−2^ (9.39)	ELYS	N
				0.861 (39.3)	ASPP2	N
				2.91 × 10^−2^ (7.41)	BAX	N
	γ_i-j_	Power coefficient for an interaction when the synthesis rate of protein i is modified by protein j. (protein-specific parameters)	–	−0.298 (9.31)	pNF-κB |- cIAP1	N
				1.85 (11.0)	BAX < - pNF-κB	N
				0.768 (8.36)	BCL2 < - pNF-κB	N
				1.0 (−^a^)	pSTAT3 < − IRAK4 VDAC1 < - pJNK ASPP2 < - ELYS	N
				1.33 (20.9)	BCL2 < - pSTAT3	N
				−4.16 (8.38)	BCL2 |- VDAC1	N
				−2.30 (30.3)	pSTAT3 |- pJNK	N
				−9.55 (16.2)	BAX |- BAX	N
				−6.10 (12.6)	ELYS |- ELYS	N
	E_i_x_	Effect of treatment x on the synthesis rate of protein i (drug-specific parameters)	–	0.960 (1.76)	IRAK4 < - B/P/B + P (*Sti*_*X*_)	Y
				−0.967 (0.503)	cIAP1 |- B (*Inh*_*B*_)	Y
				2.22 (12.2)	ELYS <- P (*Sti*_*P*_)	Y
				1.0^b^ (−^a^)	pJNK <− B + P (*Inh*_*B* + *P*_)	Y
Cell cycle and apoptosis	k_xx_	The 1st-order transition rate constant between cell cycle phases or apoptosis stages (cell-specific)	h^−1^	4.48 × 10^−2^ (8.60)	G_0_/G_1_ to S (k_12_)	N
				0.129 (7.25)	S to G_2_/M (k_23_)	N
				8.07 × 10^−2^ (5.78)	G_2_/M to G_0_/G_1_ (k_31_)	N
				2.18 × 10^−3^ (15.6)	Live to apoptotic (k_ap_) in the absence of treatment	N
				1.97 × 10^−2^ (0.938)	Mitosis-arrested to polyploid (k_pl_)	N
	N_max_	The capacity of cell density	–	7.43 (14.6)	Fold increase compared to seeding density in 6-well plates	N
	live_0_	The cell number at the beginning of the study		2.0 × 10^5^ (fixed)	Seeding density in 6-well plates	N
Integrated protein-based cell cycle and apoptosis	k_xx_	The transition rate constant between cell cycle phases or apoptosis stages (cell-specific)	h^−1^	2.39 × 10^−2^ (2.01)	Normal G_2_/M to mitotic arrested (k_ma0_)	N
				0.111 (8.22)	Mitotic arrested to apoptosis (k_apm0_)	N
	γ_i_	Power coefficient for protein i to modify a transition rate constant (protein-specific)	–	−0.532 (0.81)	cIAP1 inhibits k_ap_	N
				−7.72 × 10^−2^ (13.7)	cIAP1 inhibits k_apm0_	N
				0.208 (45.8)	BAX stimulates k_apm0_	N
				3 (−^a^)	ELYS stimulates k_ar_	N

^a^Value was fixed

^b^The combination (B + P) inhibits the degradation of pJNK completely

PTX mediates effects on cell cycle progression that can promote apoptosis. The nucleoporin/kinetochore protein ELYS, which is required for cell division, was selected as representative of a group of proteins that share similar temporal expression patterns in response to PTX. *GO* analysis, using the hypergeometric test with Benjamini-Hochberg correction, annotates this group of proteins as spindle-related (*p* < 0.01) or as regulating mitosis (*p* < 0.05). BRP alone had no effect on ELYS expression, but PTX or BRP/PTX exposure increased ELYS 1.5-fold over 24 h (Fig. 3i), followed by a return to baseline, likely because of PTX activation of spindle assembly checkpoint signals and delayed degradation of mitotic proteins [47]. Stalled mitosis, as suggested by the ELYS proteomic response, could activate the p53-mediated intrinsic apoptosis pathway [48]. Notably, the apoptosis-stimulating p53-binding protein ASPP2, selected as representative of regulators of apoptosis during a stalled mitosis, showed a similar expression pattern to ELYS in response to PTX or BRP/PTX (Fig. 3j). Supplementary Table S2 provides interaction details among these 10 proteins, along with literature-supported evidence, and Table 1 shows estimated model parameters for these proteins.

Proteomic data for each of the quantified proteins in treatment groups were normalized to their cognate protein in the vehicle control at the corresponding time point. This strategy was chosen because proliferating cells undergo changes as they approach confluency and contact inhibition, for which this approach would account. However, because some treatment groups reached confluency at different rates, we compared this approach to normalization of proteins in each group by their pretreatment time zero value. Supplementary Fig. S3 shows the temporal expression profiles for select proteins in Fig. 3 re-normalized in this manner. For most proteins, temporal regression changes in the control group were small compared to those in the drug-treated groups. Exceptions were BAX and ASPP2, in which the control group data was relatively similar in magnitude to the drug-treated groups. Nonetheless, the temporal response profiles remained similar between the two methods, despite some differences in the magnitude.

### Modeling combined effects of BRP/PTX on cell cycle distribution and apoptosis

**Fig. 4:**
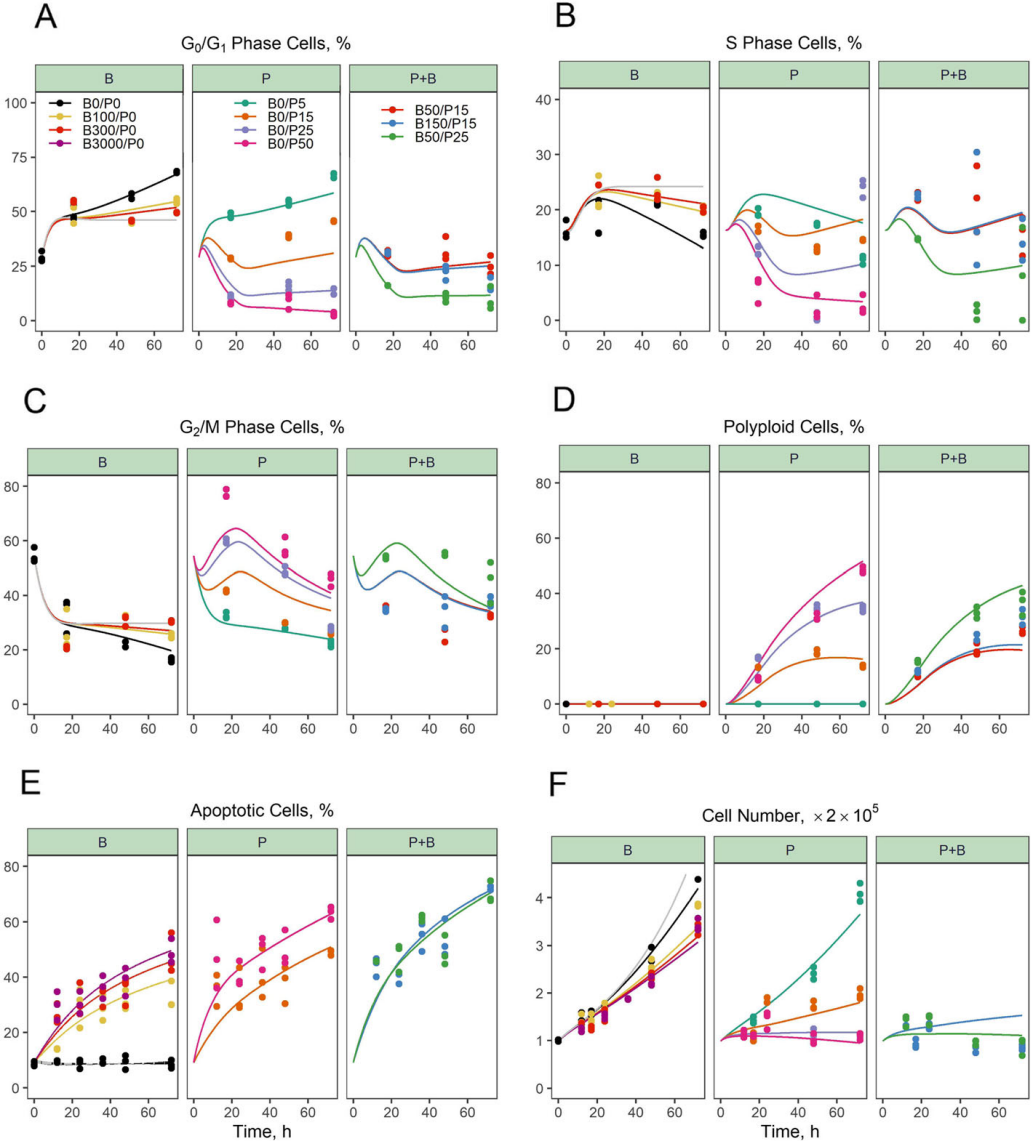
Temporal profiles of cell cycle distribution, proliferation, and apoptotic progression during birinapant/paclitaxel exposure. PANC-1 cells were exposed to vehicle, 5–50 nM PTX (P), 100–3000 nM BRP (B) or the indicated BRP/PTX (Bx/Py) combinations for up to 72 h. **a-d** Percentage of cells in G0/G1, S, and G2/M phases, and polyploid cells; **e** percentage of total apoptotic cells, consisting of early-stage (annexin^+^/7-AAD^−^) plus late-stage (annexin^+^/7-AAD^+^) cells; **f** total cell number over time. BRP alone induced apoptosis without perturbing the cell cycle drastically. PTX > 5 nM induced a transient increase in G2/M phase, followed by the accumulation of polyploid cells. Compared to PTX alone, the BRP/PTX combination decreased cells in G2/M phase and polyploid cells, and increased apoptosis. Symbols represent experimental observations (*n* = 3) and solid lines represent model-fitted profiles. The solid grey line with no symbols shows the simulated profile for the control group assuming no growth limitation

Because the proteomic analysis suggested that drug treatment perturbed mitosis, drug effects on both cell cycle distribution and apoptosis were measured over 72 h for single- and combined drug exposures. Data for the vehicle control group showed a degree of cell cycle synchronization at the initiation of drug exposure (T0) when the cell cycle distribution was first evaluated. Figure 4a-c shows a relatively high fraction of G2/M phase cells at T0 that declined over the first 17 h, whereas the G0/G1 and S phase populations rose. The partial synchrony likely resulted from plating cells from nearly-confluent cultures approx. 18 h before treatments were started. A substantive population of quiescent G0 cells may have existed at the time of plating and resumed cycling in a relatively synchronous fashion. From 17 to 72 h, a time-dependent increase of cells in G_0_/G_1_ and a decrease in S and G_2_/M cells were observed in the vehicle control group, suggesting normal progression to contact inhibition.

The cell cycle component of the model in Fig. 1b was used initially to characterize unperturbed PANC-1 cell proliferation. The cycle phase transition rate constants k_12_, k_23_, and k_31_ determine the steady-state distribution of cells in G0/G1, S, and G2/M. Figure 4a-c shows the percentage of cells in each phase if the capacity limitation of cell division, representing contact inhibition as cells approach confluency, was not implemented in the model. After 72 h of unperturbed growth, the maximum live cell number had increased to a capacity limit of 7.43-fold (Table 1, N_max_) from the beginning of the experiment, corresponding to 1.48 × 10^6^ cells/9.6cm^2^ well for 6-well plates. As the density of live cells increased, there was a gradual accumulation of cells in G_0_/G_1_ phase (Fig. 4a) as the G_0_/G_1_- to S-phase transition was prolonged. This natural inhibition of cell growth was modeled using the Gompertz function [38] as I_0_ = ln(N_max_ · *live*_0_) − ln(live) and was multiplied by k_12_, the first-order rate constant for the G_0_/G_1_- to S-phase transition. The doubling time of PANC-1 was approximated as $\frac{1}{{\mathrm{k}}_{12}\cdotp {\mathrm{I}}_0}+\frac{1}{{\mathrm{k}}_{23}}+\frac{1}{2\cdotp {\mathrm{k}}_{31}}$, and increased from 25.1 to 53.6 h over 72 h, which is consistent with literature reports [23, 40]. The first-order rate constant for naturally-occurring apoptosis in proliferating cells (k_ap_) was 2.18 × 10^− 3^ h^− 1^ (Table 1), corresponding to ~ 8% of apoptotic cells over 72 h, and was constant over time.

The model then was applied to evaluate the effects of BRP and PTX as single and combined treatments, and model-estimated parameters are listed in Table 1. For BRP alone, the total apoptotic cell population increased from a baseline of < 10% to approx. 50% over 72 h, whereas cell number at 72 h was diminished by only 12–30% compared to untreated controls (Fig. 4e,f), and even at concentrations > 10,000 nM BRP, the maximum reduction in cell number was only approx. 36% [22]. The mechanism of BRP is promotion of apoptosis.; it had minimal effect on the cell cycle (Fig. 4a-c) and mediated only slight inhibition of the total cell number (Fig. 4e and f). Thus, the continuing rise in cell number, despite increasing commitment to apoptosis, resulted from a temporal delay in completion of apoptosis, and the asynchronous initiation of the apoptotic process by cells treated with BRP alone. The fraction of late-stage apoptotic cells (annexin^+^/7-AAD^+^) cells increased continuously throughout the experiment, suggesting that early-stage apoptotic cells (annexin^+^/7-AAD^−^) progressed to cell death (Supplementary Fig. S4 A-B).

PTX alone elicited increasing, concentration-dependent G_2_/M phase arrest that peaked at 17 h (Fig. 4c), followed by an accumulation of polyploid cells (Fig. 4d). At 17 h, 50 nM PTX more than doubled the percentage of G_2_/M-phase cells (77.2 ± 1.48% vs. 33.4 ± 6.49% in the control group; values expressed as mean ± standard deviation; Fig. 4c and Supplementary Fig. S4). At 72 h, polyploid cells increased from 0% for controls to 48.4 ± 1.11% for 50 nM PTX (Fig. 4d). Low concentrations of PTX (5 nM), and single-agent BRP (50–300 nM), showed minimal perturbation of cell cycle distribution (Fig. 4a-d). Mitotically-arrested cells induced by PTX developed into polyploid cells with a first-order rate constant (k_pl_) of 1.97 × 10^− 2^ h^− 1^, which is slower than k_12_ (4.48 × 10^− 2^ h^− 1^), the rate constant for proliferating cell progression from G0/G1 to S-phase. Consistent with the literature, polyploid cells appeared resistant to drug-induced apoptosis [14–16], because the percentage of PTX-induced polyploid cells increased over 72 h (Fig. 4d).

Compared to PTX or BRP alone, combined BRP/PTX decreased both the percentage and number of G_2_/M phase cells (Fig. 4c and Supplementary Fig. S4D). At 17 h, combined BRP/PTX (150/15 nM) resulted in a 34.3% reduction in G_2_/M cells compared to PTX alone (3.87 ± 0.58 vs. 5.88 ± 0.97 × 10^4^ cells, *p* < 0.05 with Student’s t-test). By 72 h, the BRP/PTX combination produced a 43.7% decrease in polyploid cells (1.56 ± 0.23 vs. 2.78 ± 0.26 × 10^4^ cells, *p* < 0.05). Single-agent PTX and BRP alone both induced apoptosis, but combined BRP/PTX (e.g., 150/15 nM) increased apoptotic cells significantly at 72 h, from 49.0 ± 0.96% for PTX alone to 70.8 ± 2.32% for the combination (Fig. 4d; *p* < 0.01). In terms of mechanisms underlying these combination drug effects, the most likely explanation for the reduction of the G_2_/M population and subsequent increase in apoptosis by combined BRP/PTX is that BRP lowers the barriers to apoptosis of cells mitotically-arrested by PTX, which is consistent with its mechanism of action.

**Fig. 5:**
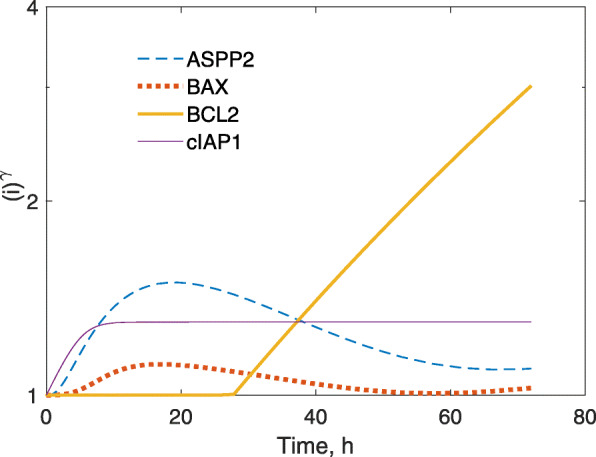
Model-simulated contribution of proteins stimulating apoptosis of mitotically-arrested cells*.* Simulation of the effect of combined 10 nM PTX and 100 nM BRP to stimulate apoptosis of mitotically-arrested cells. The simulation is based on the final protein-based cell cycle and apoptosis model of Fig. 1b, in which the k_apm_ stimulation term is regulated by ASPP2, BAX, Bcl2, and cIAP1. Each stimulation term was modeled by a power function, with the protein expression raised to a power coefficient γ, i.e., $\mathrm{sti}={\left(\mathrm{i}\right)}^{\upgamma_{\mathrm{i}}}$. The y-axis shows the strength of the k_apm_ stimulation over time, expressed as the fold change for each protein i over time to the power coefficient γ (${k}_{amp}={k}_{apm0}\cdot \mathrm{ASPP}{2}_2\cdot {cIAP}^{\gamma_{cIAP}}\cdot \frac{{\mathrm{BAX}}^{\upgamma_{\mathrm{BAX}}}}{\mathrm{Bcl}2}$, with parameter values listed in Table 1).

The following protein-mediated drug effects were integrated into the cell cycle/apoptosis regulatory model: (i) stimulation of apoptosis by a reduction in the effect of anti-apoptotic protein cIAP1 on the rate of cell transition from proliferating to apoptotic (k_ap_), (ii) incorporation of the cytostatic effect of mitotic arrest via the accumulation of kinetochore protein ELYS and its effect of increasing the transition rate (k_ma_) from cycling G_2_/M to mitotically arrested cells, and (iii) stimulation of the transition rate (k_apm_) of mitotically-arrested cells to apoptotic via the increase of pro-apoptotic proteins BAX or ASPP2, and decrease of anti-apoptotic proteins cIAP1 and Bcl2. The final model was able to capture quantitatively the drug-mediated changes in protein expression patterns, as well as effects upon cell cycle distribution and apoptosis. The BRP-mediated down-regulation of cIAP1 stimulated the transition to apoptosis (k_ap_) according to a power coefficient, represented as *k*_*ap*_ · (*cIAP*)^*γ*^, where *γ* = − 0.531, and this cIAP1 down-regulation mediated a 6.1-fold increase in the apoptosis rate. The *k*_*ap*_ · (*cIAP*)^*γ*^ rate was the same in cells exposed to combined BRP/PTX. PTX-mediated induction of ELYS-stimulated mitotic arrest, according to a mitosis arrest rate constant (k_ma_) of 2.4 × 10^− 2^ h^− 1^ at 17 h, which was only 30% of k_31_ (8.07 × 10^− 2^ h^− 1^), the rate constant for normal mitotic division, and k_ma_ was similar for BRP/PTX. The fate of mitotically-arrested cells was dependent on the apoptosis-regulating proteins ASPP2, BAX, Bcl2, and cIAP1. For combined BRP/PTX, the rate constant for arrested cells undergoing apoptosis nearly tripled over 72 h, from 0.10 to 0.29 h^− 1^, compared to 0.12 h^− 1^ for PTX treatment alone. This increase was primarily attributable to the gradual decrease in Bcl2 that was observed only with combined BRP/PTX exposure. The temporal response of ASPP2, BAX, Bcl2, and cIAP1 to k_apm_, in response to BRP/PTX (100/10 nM), is shown in Fig. 5. The down-regulation of cIAP1, and up-regulation of ASPP2 and BAX, initiated an apoptotic signal at times < 24 h. Bcl2 down-regulation then dominated the induction of mitotically-arrested apoptosis at times > 48 h. Table 1 shows the estimated model parameters. With the mechanistic components added to the model as described above, it captured the experimental data well. An additional ψ drug interaction parameter, if included in the final model, would reveal that no additional, significant, unexplained drug-drug interactions exist, i.e., that a ψ parameter for unaccounted drug interactions would be estimated as ψ = 1 if tested.

**Fig. 6:**
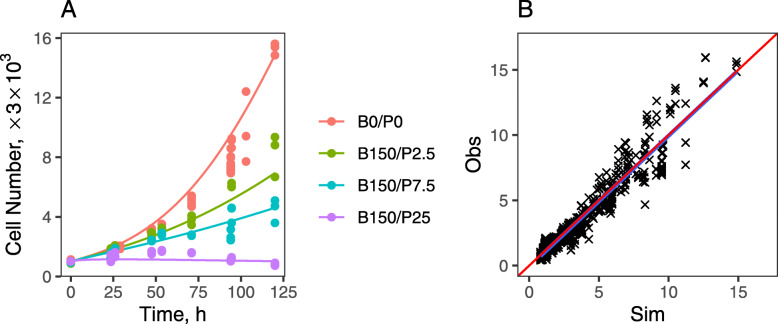
Extrapolation of the protein-based cell cycle and apoptosis model responses to drug exposure. PANC-1 cell proliferation responses to vehicle (control; P0B0) and different concentrations of BRP/PTX combinations were simulated using the final cell cycle and apoptosis model and compared with experimentally-observed values for cell proliferation over 120 h of exposure (Supplementary Fig. S1). Adjustments to the model parameters, such as increasing N_max_ (the capacity of live cells in the Gompertz function) to 30 and decreasing k_ma0_ (the rate of cycling G_2_/M cells transition to mitotically-arrested cells) to 1.67 × 10^− 2^ h^− 1^ were made to account for different culture conditions, as described in the text. **a** Representative simulations overlaid on observed cell proliferation data. **b** Simulated (Sim) vs. observed (Obs) values for 24 combinations tested experimentally. Red line represents the identity line y = x; blue line is the linear regression line. The linear regression slope was 0.92, with *R*^2^ = 0.93

The final model is based on the simultaneous analysis of all data for PANC-1 cellular responses to 100 nM BRP and 10 nM PTX, both alone and in combination. As an external qualification, model simulations were performed for different combinations of drug concentrations that were not used in construction of the model, and could then be used in validation. Observed total cell counts for 24 paired drug combinations are overlaid with the model simulations in Fig. 6a. All drug-specific parameters were converted to concentration-dependent Michaelis-Menten equations for the purpose of extrapolation. For example, the inhibition term *Inh*_*B*_, which indicates the inhibition of cIAP1 by 100 nM BRP, was converted to $In{h}_B=\frac{C_B}{C_B+I{C}_{50,B}}$, equivalent to $0.967=\frac{100}{100+I{C}_{50,B}}$, and thus *IC*_50, *B*_, the concentration of BRP causing cIAP1 to decrease to half-maximal, is 3.41 nM. By this approach, the inhibition term can be extrapolated to other concentrations as $\frac{C_B}{C_B+3.41 nM}$. Other drug-specific parameters were converted in a similar manner, except for IRAK4; its response was assumed to be dose-independent, because both single-agent- and combined BRP/PTX treatments resulted in the same IRAK4 expression profile (Supplementary Table S6). Several minor adjustments to the model were made to accommodate changes in experimental format. Cells were cultured in 96-well plates for investigation of cell growth kinetics (surface area 0.32cm^2^ per well), but the larger numbers of cells used in cell cycle and apoptosis assays required 6-well plates (surface area 9.5cm^2^ per well), leading to minor changes in proliferation kinetics. To account for the different confluence-to-seeding ratios in 6-vs. 96-wells plates, the capacity of live cells in the Gompertz function was set to a relatively high number (N_max_ = 30), and k_ma0_ was decreased by 0.3-fold for the conversion (Supplementary Table S5). All other parameters were the same as the model-estimated values in Table 1. There was good agreement between the observed cell proliferation and simulated values (Fig. 6b; *R*^2^ = 0.93), and the final proteomics-based cell cycle/apoptosis model predicted successfully the experimentally-observed synergistic inhibition of PANC-1 cell growth kinetics in response to the BRP/PTX combination.

## Discussion

Despite large-scale-, high-throughput screening efforts to identify drug combinations having enhanced therapeutic activity, such as the ALMANAC project that determined whether a pair of drugs is synergistic or antagonistic empirically [4], there exists a lack of mechanistic models for evaluating the nature and source of pharmacodynamic drug-drug interactions in oncology [49]. Consequently, there are knowledge gaps in linking the molecular mechanisms of anti-cancer drugs and treatment outcomes quantitatively. Here, a framework was developed for integrated evaluation of pharmacodynamically-based drug-drug interactions across organizational levels ranging from intracellular proteomic responses, to the status of a cell within cell cycle progression and apoptosis regulation, and to the dynamics of overall cancer cell proliferation, using the combination of BRP and PTX as proof-of-concept. As expanded here from our initial report [22], these drugs exert synergistic (supra-additive) interactions when combined. However, the mechanisms by which targeted (BRP) and non-targeted (PTX) chemotherapeutic agents act on the complex pathophysiological system of cancer cells have been unclear, along with how complex drug responses impact their overall pharmacodynamics.

In this study, a large-scale, proteome-wide analysis was employed to complement more traditional approaches for capturing drug effects on cell proliferation and apoptosis, offering new insights into diverse and complex mechanisms of drug action and interaction on a molecular level. These mechanisms, including cell cycle effects and apoptosis induction, were incorporated into the model to explain quantitatively the unidentified synergistic interaction observed with the simple cell kinetic model, based on the bioinformatic and functional annotations analyses of proteomic data (including cell cycle effects and apoptosis induction), and were tested experimentally. With these mechanisms incorporated into the model, the empirical interaction value approached 1, suggesting the model accounts mathematically for the main interactions driving the synergy. Because large numbers of temporal drug response profiles were obtained for many proteins involved in key cellular metabolism, growth, survival, and death, a clustering analysis strategy was employed to group proteins based upon their expression dynamics. Within these clusters, it was possible to identify groups of proteins linked to key biological functions, thus increasing confidence in exploring specific mechanism(s) of drug interaction. An essential element of the approach was linking quantitative information on protein dynamics to drug effects upon cell cycle, proliferation, and apoptosis by means of mathematical modeling of these cellular processes according to response networks. The final mechanistic model not only captured experimental data reflecting the drug-drug interaction, but also, through model simulations, generated new hypotheses as to how cellular protein expression profiles modulate cellular phenotypes. For example, simulations suggest that apoptosis-regulating proteins are associated with stimulating death in mitotically-arrested cells (Fig. 5). The decrease of Bcl2 from 48 to 72 h appears to be a main driver forcing PTX-arrested cells into apoptosis when combined with BRP. Therefore, a Bcl2 inhibitor could be proposed as an alternative option in combination with PTX. This approach could be extended to explore new pharmacological targets (e.g., Bcl2 inhibition) in silico to generate hypotheses identifying novel combination regimens. To develop this quantitative drug interaction modeling strategy, the interaction between paclitaxel and birinapant was first characterized by employing an empirical interaction term in a cell growth kinetic model that assumed additive cytotoxic effects of PTX and BRP, and enabled the model to quantify the degree to which observed data for drug combinations are sub- or supra-additive. This approach identified quantitatively that combined BRP/PTX is synergistic in terms of cell growth. To investigate the mechanisms of drug action underlying the apparent synergy between PTX and BRP, integrated analyses of the temporal changes in cell cycle, apoptosis, and protein expression were conducted, and all data were integrated quantitatively via pharmacodynamic modeling. Zhu el al. investigated the synergistic interaction between GEM and BRP by developing a mathematical model that hypothesized BRP potentiates GEM-induced S-phase arrest through an extended response network involving cell cycle regulation, DNA damage response, DNA repair, apoptosis, NF-κB, and mitogen- activated protein kinase (MAPK)-p38 signaling [25, 29]. We hypothesized PTX would exert stronger, more rapid, and more direct apoptotic drive than GEM, and thus better synergize with BRP. The previous approach was therefore extended to capture data for mechanisms such as mitotic arrest and emergence of a drug-resistant polyploid cell population, and was supplemented with large-scale, comprehensive proteomic analysis to provide the relevant temporal protein expression profiles that would inform as to the activity of cellular response networks. Based upon the data and modeling, a cellular response network was developed. In the model, the fate of cells mitotically-arrested by PTX was sensitive to the balance of pro-apoptotic (e.g., ASPP2 and BAX) and anti-apoptotic (e.g., cIAP1 and Bcl2) signals. The rationale for this model feature is that transcriptional activity is inhibited temporarily during mitosis, the intracellular protein synthesis rate is reduced, and therefore cells would be prone to degradation of their intracellular proteins [50]. The addition of BRP to PTX would promote proteasome-mediated degradation of cIAP [18], contributing to the anti-apoptotic signal, and providing a molecular mechanism by which BRP potentiates the overall apoptotic effect. Prolonged mitosis also leads to DNA damage and p53 induction [51], which potentially induces apoptosis after mitotic slippage. ASPP2 is an activator of the pro-apoptotic function of P53 [52], and therefore it is associated in the cell cycle and apoptosis model with apoptosis stimulation. PANC-1 cells harbor homozygous mutant P53 (R273H), which has a dominant negative activity compared to wild-type p53, and does not bind to ASPP2 [23]. However, the observed induction of ASPP2 suggests a possible p53-independent apoptotic signal, possibly mediated by ASPP2 binding to other members of the p53 family, such as p63 and p73 [52]. Because experimental data (Fig. 4) showed that expression of ASPP2 was driven primarily by PTX, and that BAX and cIAP1 were driven primarily by BRP, the model was simplified to assume those pathways were regulated only by those agents. Reducing model complexity in this way also contained the expansion of the number of protein responses necessitating experimental validation by orthogonal means, such as western blot analysis. Because the data show that activity of the pJNK-VDAC1-Bcl2 axis responded differently to combined BRP/PTX than to either of single agents, it is reasonable to hypothesize that BRP/PTX synergy likely originates from that axis. Modeling and simulations show that the contribution of Bcl2 was most prominent, especially after 48 h of exposure, and this is reflected in the data that shows combined BRP/PTX mediated a delayed but strong decline in Bcl2 (Fig. 3j).

Extension of this modeling by simulation permits the creation of testable hypotheses. For example, Bcl2, as a member of the BCL-2 protein family, regulates outer mitochondrial membrane permeability and intrinsic apoptosis [53, 54]. It prevents the oligomerization of BAX and BAK, preventing the release of cytochrome C and SMAC from the mitochondria to the cytosol. Venetoclax, the first-in-class Bcl2 inhibitor, was approved by the US FDA to treat chronic lymphocytic leukemia in 2016. Bcl2 expression is correlated with the metastatic potential of PDAC cell lines [55], and although the prognostic significance of Bcl2 up-regulation in PDAC patients is still controversial [56], the Bcl2/Bcl-xL inhibitor ABT-737 was reported to enhance PTX-induced cell death in PDAC cell lines [57, 58]. The final birinapant-paclitaxel computational model suggests a novel and possibly key role of Bcl2 in mediating the synergistic BRP/PTX interaction in PANC-1 cells, and it is reasonable to propose further investigation into whether Bcl2 inhibitors might enhance therapeutic responses of PTX.

Quantitative system pharmacological models are emerging as important tools in cancer reseach [59], and most models are fit-for-purpose [60]. Here we present a small system model that was developed using a pancreatic cancer cell line in order to explore and quantify the pharmacodynamic interactions between PTX and BRP on multiple scales, over a comparatively short period of time, in terms of effect upon proteome expression, cell cycle distribtuion, apoptosis, and overall cell proliferation. A large-scale proteomic analysis informed selection of the proteins included in this model, which were chosen as representatives of the key, differentially-expressed pathways. The model developed is a highly simplified version of a complex biological system, yet it is relavent to the interactions between PTX and BRP, based also upon prior knowledge from protein interaction literature reports. Such simplification makes more tractable the problem of integrating very large proteomic data sets into models that maintain a high degree of identifiability; these large data sets tend to be sparse in the level of experimental detail necessary to capture time- and concentration-dependent processes affecting key nodes in the model, which must therefore be obtained by alternative, orthogonal, and time-consuming experimental techniques. Because combined BRP/PTX exerted greater-than-additive inhibition of cell proliferation, represented by the cell kinetic model as unaccounted synergy in the form of parameter ψ, we explored additional mechanisms of action on cell cycle and proteomics. However, the proteomic data provided many more additional leads as to drug interaction mechanisms than could be explored reasonably. In our model, proteins responding only to the combined drugs were included as potential key mediators of the observed synergy. For example, the elevation of VDAC1 by the BRP/PTX combination could not be explained as the result of exposure to BRP or PTX alone; neither individually altered VDAC1 expression. We reported previously that BRP/PTX exposure of PANC-1 cells results in a metabolic transition from glycolysis to oxidative phosphorylation [22]. Because VDAC1 is essential in maintaining mitochondrial permeability and transporting ATP during mitochondrial respiration [35], the increase in VDAC1 might serve as an indicator of a transition to mitochondrial oxidative phosphorylation. Other drug interaction mechanism(s) might exist, but were not required in the model to explain the current data. For the proteins included in the model, the drug-mediated protein turnover process was modeled as a first-order degradation process. The estimated values for the half-times of the proteins quantified by the proteomic workflow, such as ASPP2, BAX, ELYS, IRAK4, and VDAC1, ranged from 48 min to 24.8 h, in good agreement with their half-life ranges of 45 min to 22.5 h as quantified using YFP-tagged proteins and fluorescence recovery after photobleaching [61]. This consistency with prior data increases confidence in the final structural model, estimated drug- and system-specific parameters, and the newly-generated hypotheses for the mechanisms underlying the synergistic effects of combined BRP/PTX exposure in PANC-1 cells.

The modeling approaches described here can be applied readily to other drug combinations, and extended to modeling in higher detail more complex protein interaction networks. The cell growth kinetic model is applicable to other combinations of cytotoxic drugs. Most obvious is its application to drugs that target alternative nodes in the apoptotic pathways, such as cFLIP inhibitors, and other taxanes such as docetaxel and cabazitaxel. However, any drug combinations with quantifiable pharmacodynamic endpoints are amenable. The simplicity afforded by this modeling approach is that the minimum data required is drug time- and concentration-dependence on efficacy targets such as cell viability or proliferation. The more complex multi-scale mathematical network model for cell cycle and apoptosis involved a simplified protein interactions model, which is highly amenable to the integration of large-scale ‘omics’ data, that was linked into a somewhat more conventional pharmacodynamic cell cycle/apoptosis model, that was selected to represent key details of the ultimate cellular targets of interest, based on the mechanisms of action of the two drugs. Thus, the model is ‘fit for purpose’, balancing complexity against the primary objectives of the study. The use of clustering of the proteomics data based upon temporal response patterns enabled us to distill from this rich data set the responses of key functional networks of interest for closer bioinformatic analysis and attention and, in future studies, holds the potential to reveal cascading response networks within drug-perturbed cellular systems.

## Conclusions

A multi-scale pharmacodynamic modeling framework was developed to investigate the potential sources of synergy between an apoptosis-promoting drug, birinapant, and an apoptosis-inducing drug, paclitaxel. This framework leverages advances in the science of comprehensive, quantitative proteomic data acquisition to provide new approaches to investigate the pharmacodynamic mechanisms of drug-drug interactions.

## Supplementary information

**Additional file 1.**

## Data Availability

The code for the models in this paper, and datasets for the subset of proteins analyzed, are available at GitHub (https://github.com/niujin2013/Proteomics_modeling_Panc1). The entire mass spectrometry proteomics data has been deposited in the ProteomeXchange Consortium via the PRIDE partner repository [62] with the dataset identifiers PXD007890 and10.6019/PXD007890.

## Abbreviations

7-AAD: 7-aminoactinomycin D
ABX: Abraxane
ALMANAC: A Large Matrix of Anti-Neoplastic Agent Combinations
ASPP2: apoptosis-stimulating of p53 protein 2, TP53BP2
BAX: Bcl2-associated X protein
Bcl-2: B-cell lymphoma 2
BRP: birinapant
CDKN2A: Cyclin dependent kinase inhibitor 2A
cIAP1: cellular inhibitor of apoptosis protein 1
DAVID: Database for Annotation, Visualization and Integrated Discovery
DMSO: dimethylsulfoxide
EDTA: ethylenediaminetetraacetate
ELYS: protein ELYS is encoded by gene AHCTF1
GEM: gemcitabine
IRAK4: interleukin 1 receptor associated kinase 4
(p) JNK: (phosphorylated) c-Jun N-terminal kinase (MAPK 8)
(p) NF-κB: (phosphorylated) nuclear factor kappa B
PE: phycoerythrin
PI: Propidium iodide
PTX: paclitaxel
SMAC: second mitochondria-derived activator of caspases
SRB: Sulforhodamine B
(p) STAT3: (phosphorylated) signal transducer and activator of transcription 3
TNFα: Tumor necrosis factor α
TP53: Tumor protein P53
VDAC1: voltage dependent anion channel 1
