# Supplementary material for: Pharmacodynamic modeling of synergistic birinapant/paclitaxel interactions in pancreatic cancer cells
Source: BMC Cancer. 2020 Oct 23;20:1024. doi: 10.1186/s12885-020-07398-9 (PMC7583190; doi:10.1186/s12885-020-07398-9)
Supplement: Supplementary file 1 — Additional file 1. [file 12885_2020_7398_MOESM1_ESM.docx]

Supplemental Data

Pharmacodynamic modeling of synergistic birinapant/paclitaxel interactions in pancreatic cancer cells

Jin Niu^1^, Xue Wang^2,3^, Jun Qu^1,2,3^, Donald E. Mager^1^, Robert M. Straubinger^1,3,4,*^

**Author Affiliations**

^1^ Department of Pharmaceutical Sciences, University at Buffalo, State University of New York, Buffalo, New York, USA

^2^ Department of Cell Stress Biology, Roswell Park Cancer Institute, Buffalo, New York, USA

^3^ New York State Center of Excellence in Bioinformatics and Life Sciences, New York, USA

^4^ Department of Pharmacology and Therapeutics, Roswell Park Cancer Institute, Buffalo, New York, USA

**^*^ Corresponding Author:**

Department of Pharmaceutical Sciences
University at Buffalo, State University of New York
Buffalo, New York 14214
rms@buffalo.edu

**Table of Contents**

[Supplemental Tables 2](#_Toc50546604)

[Table S1. Equations for cell growth kinetic model of cellular responses to paclitaxel and birinapant. 2](#_Toc50546605)

[Table S2. Prior knowledge/evidence supporting protein interaction model. 3](#_Toc50546606)

[Table S3. Equations for cell cycle and apoptosis model based on large-scale proteomic analysis. 4](#_Toc50546607)

[Table S4. Parameter estimation for the cell growth kinetic model. 8](#_Toc50546608)

[Table S5. Correction for polyploid cell number in the SRB assay. 9](#_Toc50546609)

[Table S6. Transformations of parameters used to extrapolate the quantitative relationships between drug concentrations and cell dynamics. 10](#_Toc50546610)

[Supplemental Figures 11](#_Toc50546611)

[Figure S1. Birinapant/paclitaxel effects on PANC-1 cell proliferation. 11](#_Toc50546612)

[Figure S2. Clustering of temporal protein expression responses to birinapant/paclitaxel treatment. 13](#_Toc50546613)

[Figure S3. Effect of normalization method upon on protein expression profiles. 14](#_Toc50546614)

[Figure S4. Birinapant/paclitaxel effects on cell cycle distribution and apoptosis. 15](#_Toc50546615)

[References 17](#_Toc50546616)

# Supplemental Tables

## Table S1. Equations for cell growth kinetic model of cellular responses to paclitaxel and birinapant.

In the model schematic in Figure 1A of the parent publication, $N$ is the cell number normalized to the initial cell number immediately before treatment (T=0) and $k_{G}$ is the first-order net growth rate constant for PANC-1 cells. For the remaining parameters, subscripted X, represent either paclitaxel (P) or birinapant (B); $K_{\max}$is the maximum killing rate constant, C is the drug concentration in the culture medium, $\mathrm{KC}_{50}$ is the concentration that induces 50% of the killing capacity, $\gamma$ is the Hill coefficient, $S_{i}$ (where i=1, 2, 3) represents hypothetical signal transduction compartments that introduce a time-delay in the downstream pharmacodynamic response, and $\tau$ is the mean transit time between each compartment. We assumed that the killing signals of paclitaxel and birinapant are additive, and that the drug combination would change the sensitivity to each drug. Therefore, the interaction term $\Psi$ was multiplied with both $\mathrm{KC}_{50}$ parameters when analyzing drug exposure for the drugs combined, and represents the departure of the data from an additive interaction.

| Ordinary differential equations (ODEs) | Initial conditions (ICs) |  |
| --- | --- | --- |
| $\frac{\mathrm{dN}}{\mathrm{dt}}=N\cdot k_{G}-N\cdot\sum_{X} S_{3X}$ | $1$ | Eq1 |
| $\frac{dS_{1X}}{\mathrm{dt}}=\frac{1}{\tau_{X}}\cdot\left( \frac{K_{\mathrm{maxX}}{C_{X}}^{\gamma_{X}}}{{C_{X}}^{\gamma}+\left( {\Psi\cdot KC}_{50X} \right)^{\gamma_{X}}}-S_{1X} \right)$ | $0$ | Eq2 |
| $\frac{dS_{\mathrm{iX}}}{\mathrm{dt}}=\frac{1}{\tau_{X}}\cdot\left( S_{i-1X}-S_{\mathrm{iX}} \right)$ | $0 (i=2 or 3)$ | Eq3 |
| Error Model |  |  |
| $\mathrm{Va}r_{e}=\left( \delta+\sigma\cdot N \right)^{2}$ |  |  |

## Table S2. Prior knowledge/evidence supporting protein interaction model.

| Source | Target | Direction | Reference |
| --- | --- | --- | --- |
| cIAP1 | p NF-κB p65 | Activation | [1] |
| p NF-κB p65 | BAX | Activation | [2] |
| p NF-κB p65 | Bcl2 | Activation | [3] |
| BAX | BAX | Inhibition | [4] |
| IRAK4 | p STAT3 | Activation | [5] |
| p STAT3 | Bcl2 | Activation | [6] |
| p JNK | p STAT3 | Inhibition | [7] |
| p JNK | VDAC1 | Activation | [8] |
| VDAC1 | Bcl2 | Inhibition | [9] |
| ELYS | ASPP2 | Activation | [10] |

## Table S3. Equations for cell cycle and apoptosis model based on large-scale proteomic analysis.

For the model structure shown in Figure 1B of the parent publication, equations 4-16 represent the protein interactions, characterized by indirect response models, in which, $k_{\deg_{A}}$ is the degradation rate constant for protein A. Because treatment-mediated protein expression responses are normalized to the vehicle control group at each corresponding time point, the baseline expression for each protein is 1. Thus, the production rate for protein A is equal to $k_{\deg_{A}}$, and $\gamma_{A-B}$ is the power coefficient for protein B to modify the production rate of protein A. $\mathrm{Inh}_{X}$ or $\mathrm{Sti}_{X}$ represents the inhibition or stimulation by drug X (B for birinapant, P for paclitaxel, B+P for the combination). $A_{\mathrm{fb}}$ represents the hypothetical self-feedback mechanism(s) for protein A. Equations 17-22 represent the progression of cells throgh the cell cycle or toward apoptosis. In the absence of treatment, each proliferating cell progresses through G_0_/G_1_, S and G_2_/M phases, and then divides into 2 progeny cells, according to rate constants $k_{12}, k_{23}$, and$k_{31}$, respectively. Untreated cells undergo intrinsic apoptosis with a rate constant of $k_{\mathrm{ap}}$, and apoptotic cells (Apo) eventually disappear according to the same rate. As cell density increases, nutrients and/or growth factors are consumed, which causes cells to exit cycling and remain in G_0_ phase. Therefore, a cell density-dependent inhibition I_0_, which slows G_0_/G_1_- to S phase progression gradually, is modeled with a Gompertz differential equation [11], in which parameter $N_{max}$ represents the maxiumal number of cycling cells under the culture conditions (Eq23). Production of the paclitaxel-induced kinetochore protein ELYS leads to mitotically arrested cells (M_A_) with a rate constant $k_{\mathrm{ma}}$, which is stimulated by a power function of relative ELYS abundance (Eq24). The arrested cells either (i) undergo apoptosis with a rate constant of $k_{\mathrm{apm}}$, which is modified by the expression of BAX, Bcl2, and $\mathrm{ASPP}2_{2}$, which is a delayed signal from ASPP2 (Eq14 and Eq25), or (ii) the arrested cells can also undergo mitotic slippage and ultimately form polyploid cells (PL) with a rate constant of $k_{\mathrm{pl}}$. The polyploid cells are assumed to be drug resistant, and therefore the disapperance of these cells is controlled solely by $k_{\mathrm{pl}}$. Because cell cycle analysis is based on cellular DNA content, as determined by propidium iodide (PI) staining, the sum of G_2_/M + M_A_ cells was considered for model fitting purposes to represent tetraploid cells. Cells having a DNA content higher than tetraploid were considered as polyploid.

| ODEs | ICs |  |
| --- | --- | --- |
| $\frac{dcIAP1}{\mathrm{dt}}=k_{\deg_{cIAP1}}\cdot\left( 1-\mathrm{Inh}_{B} \right)-k_{\deg_{cIAP1}}\cdot cIAP1$ | $1$ | Eq4 |
| $\frac{dpp65}{\mathrm{dt}}=k_{\deg_{pp65}}\cdot\left( cIAP1 \right)^{\gamma_{pp65-cIAP1}}-k_{\deg_{pp65}}\cdot pp65$ | $1$ | Eq5 |
| $\frac{\mathrm{dBAX}}{\mathrm{dt}}=k_{\deg_{\mathrm{BAX}}}\cdot\left( pp65 \right)^{\gamma_{BAX-pp65}}\cdot\left( \mathrm{BAX}_{\mathrm{fb}} \right)^{\gamma_{BAX-\mathrm{BAX}_{\mathrm{fb}}}}-k_{\deg_{\mathrm{BAX}}}\cdot BAX$ | $1$ | Eq6 |
| $\frac{dBcl2}{\mathrm{dt}}=k_{\deg_{Bcl2}}\cdot max\left( \left( pp65 \right)^{\gamma_{Bcl2-pp65}}, \left( pSTAT3 \right)^{\gamma_{Bcl2-pSTAT3}} \right)\cdot\left( VDAC1 \right)^{\gamma_{Bcl2-VDAC1}}-k_{\deg_{Bcl2}}\cdot Bcl2$ | $1$ | Eq7 |
| $\frac{dIRAK4}{\mathrm{dt}}=k_{\deg_{IRAK4}}\cdot\left( 1+\mathrm{Sti}_{X} \right)-k_{\deg_{IRAK4}}\cdot IRAK4$ | $1$ | Eq8 |
| $\frac{dpSTAT3}{\mathrm{dt}}=k_{\deg_{pSTAT3}}\cdot\left( \mathrm{pJNK} \right)^{\gamma_{pSTAT3-pJNK}}\cdot IRAK4-k_{\deg_{pSTAT3}}\cdot pSTAT3$ | $1$ | Eq9 |
| $\frac{\mathrm{dpJNK}}{\mathrm{dt}}=k_{\deg_{\mathrm{pJNK}}}-k_{\deg_{\mathrm{pJNK}}}\cdot pJNK\cdot(1-\mathrm{Inh}_{B+P})$ | $1$ | Eq10 |
| $\frac{dVDAC1}{\mathrm{dt}}=k_{\deg_{VDAC1}}\cdot pJNK-k_{\deg_{VDAC1}}\cdot VDAC1$ | $1$ | Eq11 |
| $\frac{\mathrm{dELYS}}{\mathrm{dt}}=k_{\deg_{\mathrm{ELYS}}}\cdot(1+\mathrm{Sti}_{P})\cdot\left( \mathrm{ELYS}_{\mathrm{fb}} \right)^{\gamma_{ELYS-\mathrm{ELYS}_{\mathrm{fb}}}}-k_{\deg_{\mathrm{ELYS}}}\cdot ELYS$ | $1$ | Eq12 |
| $\frac{dASPP2}{\mathrm{dt}}=k_{\deg_{ASPP2}}\cdot ELYS-k_{\deg_{ASPP2}}\cdot ASPP2$ | $1$ | Eq13 |
| $\frac{\mathrm{dASPP}2_{2}}{\mathrm{dt}}=k_{\deg_{ASPP2}}\cdot ASPP2-k_{\deg_{ASPP2}}\cdot ASPP2_{2}$ | 1 | Eq14 |
| $\frac{\mathrm{dBAX}_{\mathrm{fb}}}{\mathrm{dt}}=k_{\deg_{\mathrm{BAX}}}\cdot BAX-k_{\deg_{\mathrm{BAX}}}\cdot\mathrm{BAX}_{\mathrm{fb}}$ | $1$ | Eq15 |
| $\frac{\mathrm{dELYS}_{\mathrm{fb}}}{\mathrm{dt}}=k_{\deg_{\mathrm{ELYS}}}\cdot ELYS-k_{\deg_{\mathrm{ELYS}}}\cdot\mathrm{ELYS}_{\mathrm{fb}}$ | $1$ | Eq16 |
| $\frac{dG_{0}G_{1}}{\mathrm{dt}}={2\cdot k}_{31}\cdot G_{2}M-k_{12}\cdot G_{0}G_{1}\cdot I_{0}-k_{\mathrm{ap}}\cdot\left( cIAP1 \right)^{\gamma_{cIAP1}}\cdot G_{0}G_{1}$ | ${{G_{0}G}_{1}}_{\mathrm{ini}}$ | Eq17 |
| $\frac{\mathrm{dS}}{\mathrm{dt}}=k_{12}\cdot G_{0}G_{1}\cdot I_{0}-k_{23}\cdot S-k_{\mathrm{ap}}\cdot\left( cIAP1 \right)^{\gamma_{cIAP1}}\cdot S$ | $S_{\mathrm{ini}}$ | Eq18 |
| ${\frac{dG_{2}M}{\mathrm{dt}}=k_{23}\cdot S-k}_{31}\cdot G_{2}M-k_{\mathrm{ap}}\cdot\left( cIAP1 \right)^{\gamma_{cIAP1}}\cdot G_{2}M-k_{\mathrm{ma}}\cdot G_{2}M$ | ${G_{2}M}_{\mathrm{ini}}$ | Eq19 |
| $\frac{dM_{A}}{\mathrm{dt}}=k_{\mathrm{ma}}\cdot G_{2}M-k_{\mathrm{apm}}\cdot M_{A}-k_{\mathrm{pl}}\cdot M_{A}$ | $0$ | Eq20 |
| $\frac{\mathrm{dPL}}{\mathrm{dt}}=k_{\mathrm{pl}}\cdot M_{A}-k_{\mathrm{pl}}\cdot PL$ | $0$ | Eq21 |
| $\frac{\mathrm{dApo}}{\mathrm{dt}}=k_{\mathrm{ap}}\cdot\left( cIAP1 \right)^{\gamma_{cIAP1}}\cdot\left( G_{0}G_{1}+S+G_{2}M \right)+k_{\mathrm{apm}}\cdot M_{A}-k_{\mathrm{ap}}\cdot Apo$ | $\mathrm{Apo}_{\mathrm{ini}}$ | Eq22 |
| Where, |  |  |
| $I_{0}=\ln\left( N_{\max}\cdot\mathrm{Live}_{0} \right)-\ln\left( Live \right)$  $=\ln\left( N_{\max}\cdot\left( {{G_{0}G}_{1}}_{\mathrm{ini}}+S_{\mathrm{ini}}+G_{2}M_{\mathrm{ini}} \right) \right)-\ln\left( G_{0}G_{1}+S+G_{2}M+M_{A}+PL \right)$ | | Eq23 |
| $k_{\mathrm{ma}}=\left\{ \begin{aligned} 0, if ELYS\leq1; \\ k_{ma0}\cdot\left( \mathrm{ELYS} \right)^{\gamma_{\mathrm{ELYS}}}, otherwise. \end{aligned} \right. \cdot$ |  | Eq24 |
| $k_{\mathrm{apm}}=\left\{ \begin{aligned} 0, if MA=0; \\ k_{apm0}\cdot ASPP2_{2}\cdot\left( cIAP1 \right)^{\gamma_{cIAP1}}\cdot\frac{\mathrm{BA}X^{\gamma_{\mathrm{BAX}}}}{Bcl2}, otherwise. \end{aligned} \right.$ |  | Eq25 |
| And, |  |  |
| $Total=G_{0}G_{1}+S+G_{2}M+M_{A}+PL+Apo$ |  | Eq26 |
| $Live=G_{0}G_{1}+S+G_{2}M+M_{A}+PL$ |  | Eq27 |
|  |  |  |
| $Apo\%=\frac{\mathrm{Apo}}{\mathrm{Total}}\cdot100\%$ |  | Eq28 |
| $G_{0}G_{1}\%=\frac{G_{0}G_{1}}{\mathrm{Live}}\cdot100\%$ |  | Eq29 |
| $S \%=\frac{S}{\mathrm{Live}}\cdot100\%$ |  | Eq30 |
| $G_{2}M \%=\frac{G_{2}M+M_{A}}{\mathrm{Live}}\cdot100\%$ |  | Eq31 |
| $PL \%=\frac{\mathrm{PL}}{\mathrm{Live}}\cdot100\%$ |  | Eq32 |
| **Error models:** |  |  |
| For protein expression in log2 scale: constant |  |  |
| For cell cycle or apoptosis percentage: constant |  |  |
| For total cell number: $\mathrm{Va}r_{e}=\left( \delta_{n}+\sigma_{n}\cdot Total \right)^{2}$ |  |  |

## Table S4. Parameter estimation for the cell growth kinetic model.

| **Parameter** | | **Birinapant** |  | **Paclitaxel** | |  |
| --- | --- | --- | --- | --- | --- | --- |
|  |  | Estimate | CV% | Estimate | | CV% |
| $K_{\max}, h^{-1}$ | Maximum cell killing rate constant | 1.53$\cdot{10}^{-2}$ | 18.1 | 2.33$\cdot{10}^{-2}$ | | 3.30 |
| $\mathrm{KC}_{50}$, nM | Concentration to induce 50% of maximum cell killing | 277 | 40.6 | 18.3 | | 3.52 |
| $\gamma$ | Hill coefficient | 0.823 | 13.9 | 2.73 | | 5.48 |
| $\tau$, h | Mean transit time between signaling compartments | 11.0 | 16.4 | 4.62 | | 9.72 |
|  |  | Estimate | CV% | |  |  |
| $k_{G}, h^{-1}$ | Net growth rate constant | 2.25$\cdot{10}^{-2}$ | 1.08 | |  |  |
| $\Psi$ | Interaction term | 0.690 | 3.71 | |  |  |
| $\delta$ | Intercept for variance model | 0.0788 | 16.2 | |  |  |
| $\sigma$ | Slope for variance model | 0.106 | 6.46 | |  |  |

## Table S5. Correction for polyploid cell number in the SRB assay.

|  | B300/P20 | | P20 |  |  |
| --- | --- | --- | --- | --- | --- |
| Time, h | Polyploid, %* | CF** | Polyploid, % | CF | CF_BP:CF_P |
| 0 | 0.0 | 1.00 | 0.0 | 1.00 | 1.00 |
| 24 | 23.8 | 0.81 | 24.8 | 0.80 | 1.01 |
| 48 | 35.3 | 0.74 | 38.3 | 0.72 | 1.02 |
| 72 | 34.9 | 0.74 | 39.3 | 0.72 | 1.03 |
| 96 | 29.0 | 0.78 | 33.8 | 0.75 | 1.04 |
| 120 | 18.2 | 0.85 | 26.4 | 0.79 | 1.07 |

*: the percentage of polyploid cells were based on the predictions from the cell cycle and apoptosis model.

**CF: correction factor, calculated by $\frac{true number}{apparant number}=\frac{N_{diploid}+ N_{polyploid}}{N_{diploid}+ {2\cdot N}_{polyploid}}$, assuming the size of the polyploid cells are twice as the diploid cells.

## Table S6. Transformations of parameters used to extrapolate the quantitative relationships between drug concentrations and cell dynamics.

| **Between drug concentrations and protein dynamics:** | | | | | | | | | | | |
| --- | --- | --- | --- | --- | --- | --- | --- | --- | --- | --- | --- |
| Parameter | Target | Equation | Calculation | | New parameter | | Value | | Unit | | |
| $\mathrm{Inh}_{B}$ | cIAP1 | $Inh_{B}=\frac{C_{B}}{C_{B}+ IC_{50,B}}$ | $0.967=\frac{100}{100+ IC_{50,B}}$ | | ${IC}_{50,B}$ | | 3.41 | | nM | | |
| $\mathrm{Sti}_{P}$ | ELYS | $1+\mathrm{Sti}_{P}=\frac{1}{1-Inh_{P}},$  $Inh_{P}=\frac{C_{P}}{C_{P}+ IC_{50,P}}$ | $1+2.22= \frac{10+ IC_{50,P}}{{IC}_{50,P}}$ | | ${IC}_{50,P}$ | | 4.50 | | nM | | |
| $\mathrm{Inh}_{B+P}$ | pJNK | $\mathrm{Inh}_{B+P}=\frac{C_{B+P}}{C_{B+P}+ IC_{50,B+P}}$ | $0.999=\frac{110}{110+ IC_{50,B+P}}$ | | ${IC}_{50,B+P}$ | | 0.11 | | nM | | |
| $\mathrm{Sti}_{X}$ | IRAK4 | - | - | | $\mathrm{Sti}_{X}$ | | 0.960 | | - | | |
| **Adjustments between 96 well plates and 6 well plates:** | | | | | | | | | |  |  |
| Parameter |  | | | Value for 6-well plate | | Value for 96-well plates | | Unit | | |  |
| $N_{\max}$ | Maximal cell number divided by cell number at time t=0. | | | 7.43 | | 30 | | - | | |  |
| $k_{ma0}$ | Rate constant for cells to progress from G_2_/M to mitotically arrested state. | | | 2.39×10^-2^ | | 1.67×10^-2^ | | h^-1^ | | |  |

# Supplemental Figures

## Figure S1. Birinapant/paclitaxel effects on PANC-1 cell proliferation.

PANC-1 cells were cultured in 96-well plates and exposed to paclitaxel (PTX), birinapant (BRP), or combined (24 pairs of varying-ratio combinations) for up to 120h. Cell viability was measured by the SRB assay. Cell growth kinetics are shown for exposure to: (A) BRP as a single agent, (B) PTX as a single agent, or to (C-H) fixed combinations of 2.5, 7.5, 20, 25, 30, 60nM PTX *(P)* with various concentrations of BRP *(B)* ranging from 15 to 1000nM. The x axis represents exposure durations of 0 to 120h. The y axis the represents cell viability ratio, which is normalized to the mean value at T=0. The inset legends indicate the drug (B or P) and its concentration, *i.e.*, P0B0 represents the vehicle control, whereas P7.5B100 represents combined 7.5nM PTX and 100nM BRP. Symbols represent experimental observations (n=3), and solid lines represent model fitting results.

| 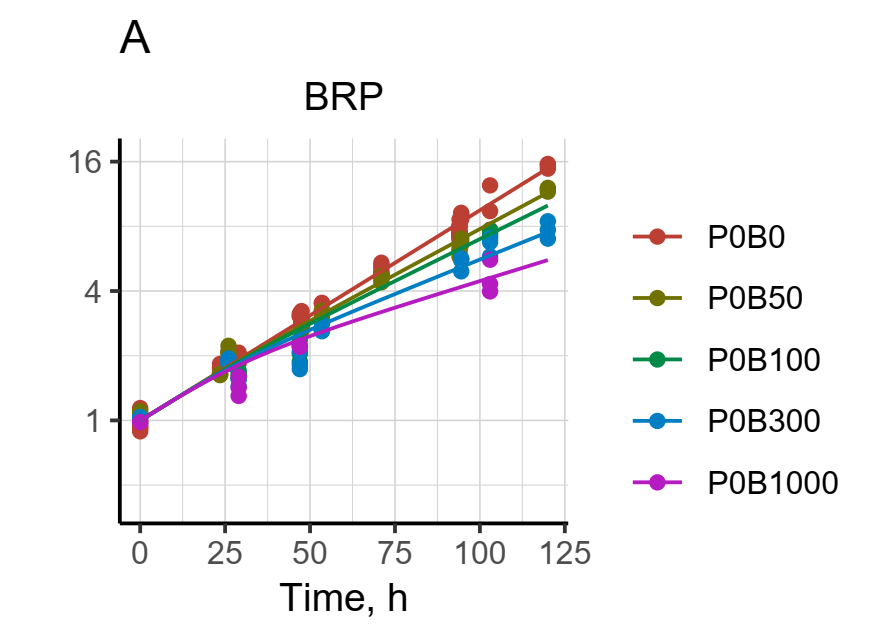 | 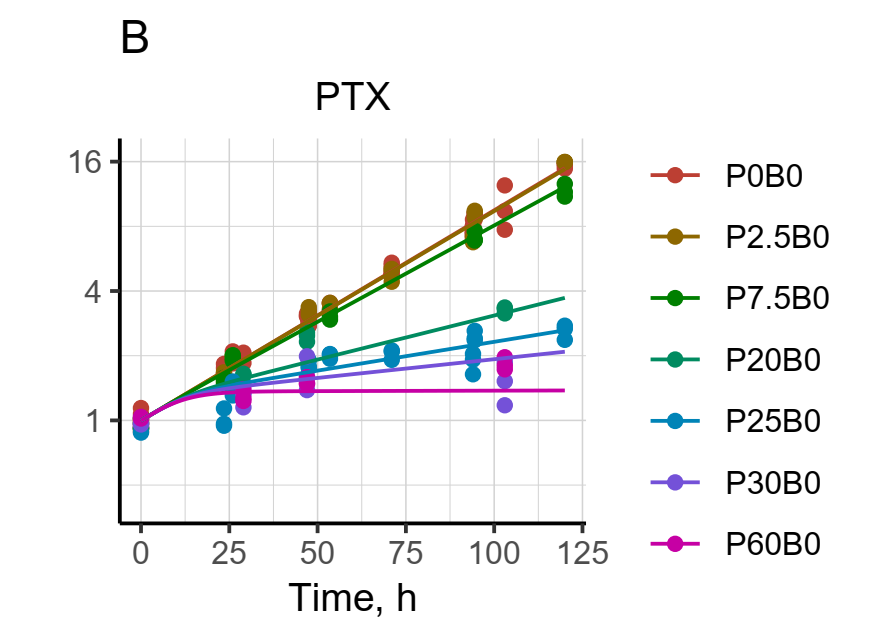 |
| --- | --- |
| 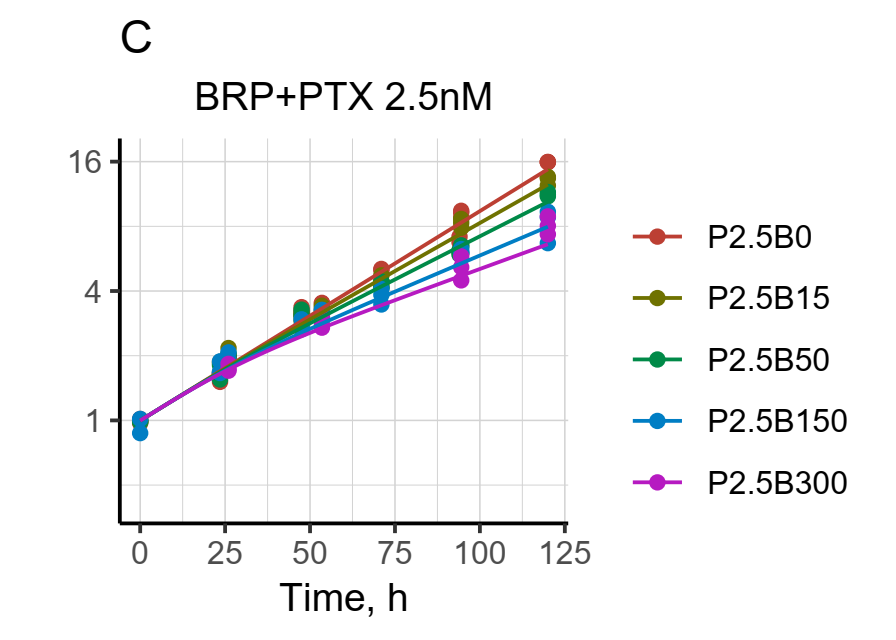 | 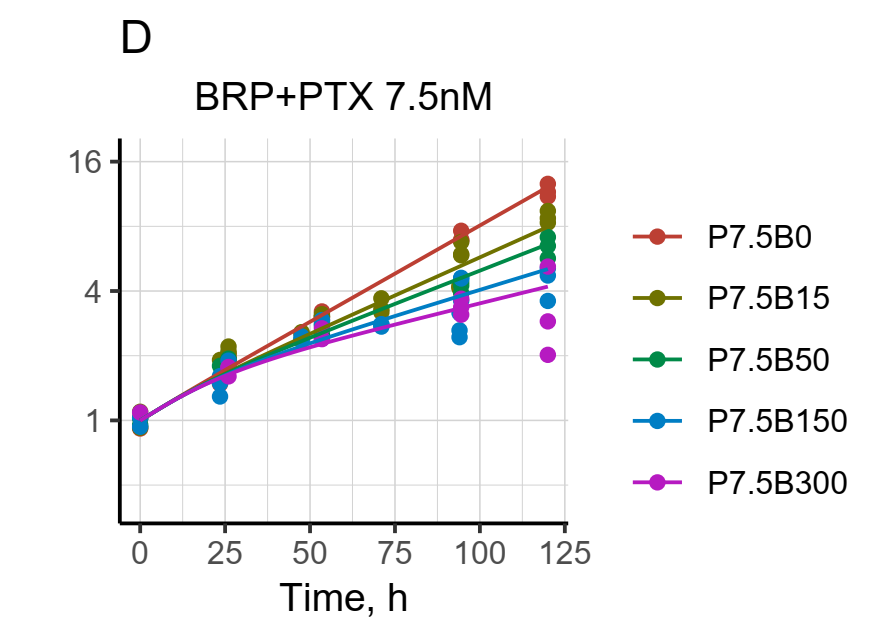 |
| 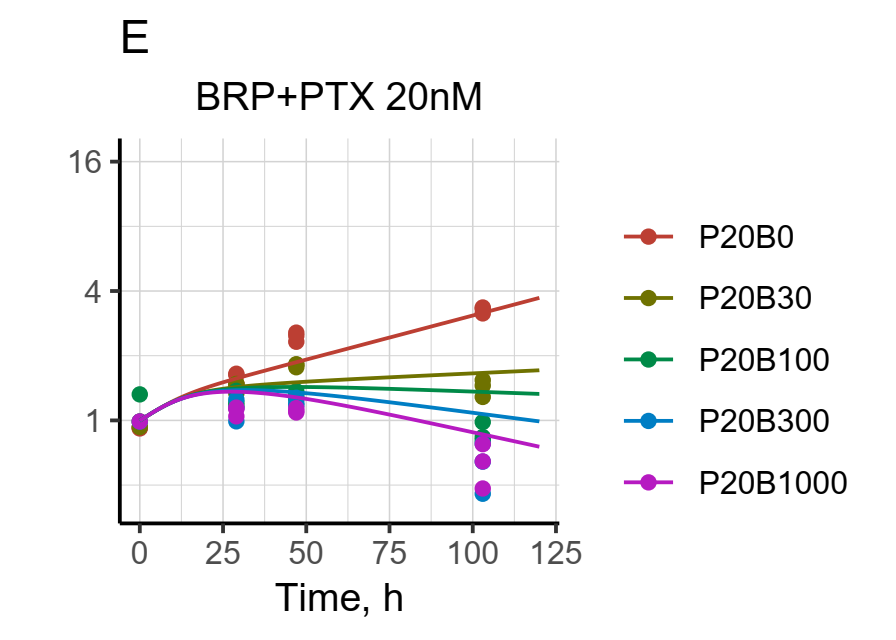 | 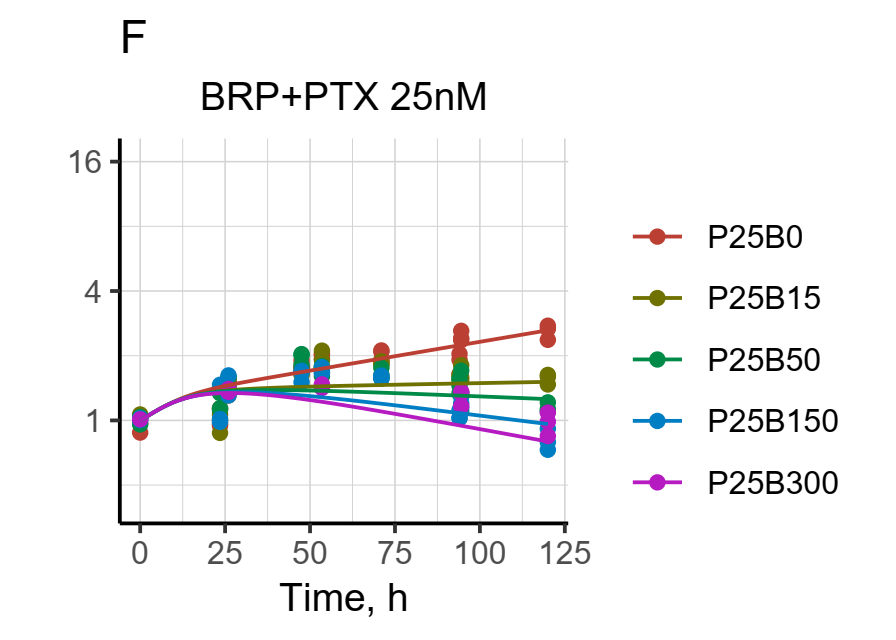 |
| 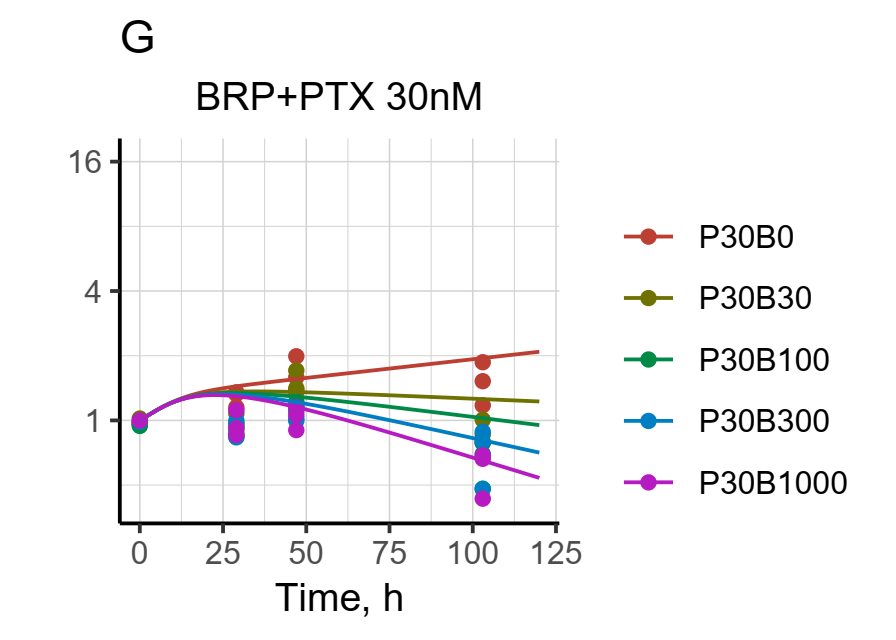 | 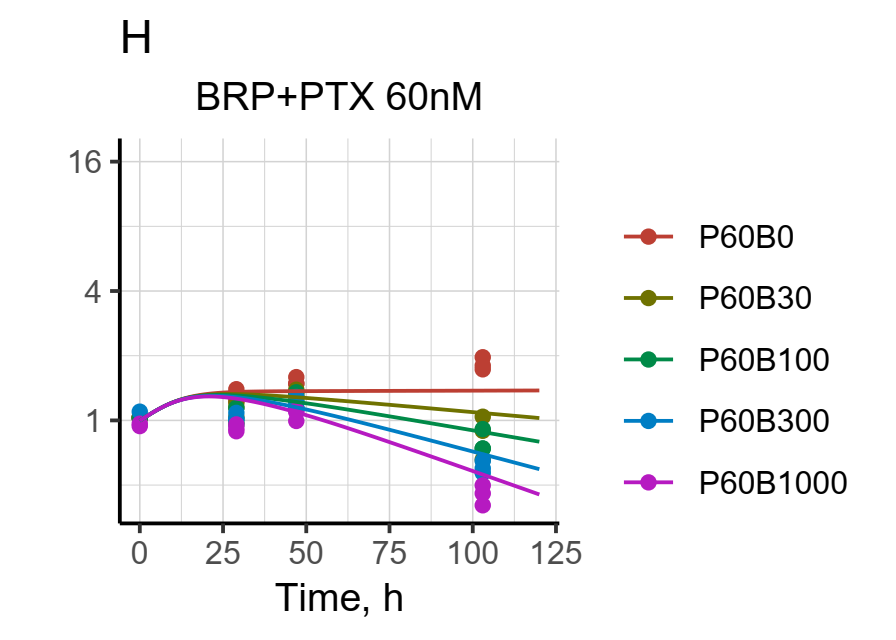 |

## Figure S2. Clustering of temporal protein expression responses to birinapant/paclitaxel treatment.

Quantitative proteomic analysis was performed using the IonStar workflow [12] on PANC-1 cells exposed to vehicle (0.1% DMSO), or 100nM birinapant and 10nM paclitaxel, alone or combined, for 6, 24, 48 and 72h. The k-means clustering algorithm of the STEM software application [13] was applied to quantitative proteomics data capturing the treatment-mediated changes in protein expression. The maximum deviation of experimental data from a cluster’s mean was calculated as a function of the number of data clusters. Based upon this analysis, a total of 9 clusters was selected for subsequent modeling.


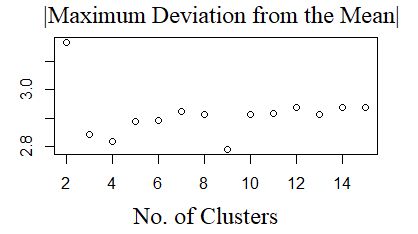


## Figure S3. Effect of normalization method upon on protein expression profiles.

Quantitative proteomic analysis was conducted for PANC-1 cells exposed to vehicle control (0. 1% DMSO) and 100nM birinapant and 10nM paclitaxel for 0, 6, 24, 48 and 72h. In the main manuscript, each protein was normalized to the same protein in the control group, so as to account for the fact that cultures of proliferating cells undergo protein-level changes as they approach contact inhibition. Here, expression of select proteins was normalized to its own value at time zero. In the majority of cases, the temporal changes in the control group were small compared to changes in the drug-treated groups. Exceptions were ASPP2 and BAX. However, for all of the proteins, the temporal response proteins remained very similar to those shown in the parent manuscript.

## Figure S4. Birinapant/paclitaxel effects on cell cycle distribution and apoptosis.

PANC-1 cells were cultured in 6-well plates and exposed for up to 72h to vehicle (0.1% DMSO), or 50-150nM BRP and 15-50nM PTX, alone or in combination. (A, B) Percent of cells in late-stage apoptosis (AnnexinV^+^/7-AAD^+^) after BRP/PTX treatment. (C) Percent of cells in G_0_/G_1_, S, G_2_/M phases and polyploid after 17h drug exposure. (D, E) Total G_2_/M phase- and polyploid cells after 17h exposure to PTX, alone or combined with BRP. (F) Percentage of all apoptotic (AnnexinV^+^) cells after 72h exposure to PTX or combinations. Data depicted are mean ± SD; *, p<0.05 by Student’s t-test.

| A | B |
| --- | --- |
|  | |
| C   | D   |
| E   | F   |

# References

1. Vince JE, Wong WW, Khan N, Feltham R, Chau D, Ahmed AU, Benetatos CA, Chunduru SK, Condon SM, McKinlay M *et al*: **IAP antagonists target cIAP1 to induce TNFalpha-dependent apoptosis**. *Cell* 2007, **131**(4):682-693.

2. Shou Y, Li N, Li L, Borowitz JL, Isom GE: **NF-kappaB-mediated up-regulation of Bcl-X(S) and Bax contributes to cytochrome c release in cyanide-induced apoptosis**. *J Neurochem* 2002, **81**(4):842-852.

3. Catz SD, Johnson JL: **Transcriptional regulation of bcl-2 by nuclear factor kappa B and its significance in prostate cancer**. *Oncogene* 2001, **20**(50):7342-7351.

4. Reyna DE, Gavathiotis E: **Self-regulation of BAX-induced cell death**. *Oncotarget* 2016, **7**(41):66326-66327.

5. Kelly PN, Romero DL, Yang Y, Shaffer AL, 3rd, Chaudhary D, Robinson S, Miao W, Rui L, Westlin WF, Kapeller R *et al*: **Selective interleukin-1 receptor-associated kinase 4 inhibitors for the treatment of autoimmune disorders and lymphoid malignancy**. *J Exp Med* 2015, **212**(13):2189-2201.

6. Real PJ, Sierra A, De Juan A, Segovia JC, Lopez-Vega JM, Fernandez-Luna JL: **Resistance to chemotherapy via Stat3-dependent overexpression of Bcl-2 in metastatic breast cancer cells**. *Oncogene* 2002, **21**(50):7611-7618.

7. Lim CP, Cao X: **Serine phosphorylation and negative regulation of Stat3 by JNK**. *J Biol Chem* 1999, **274**(43):31055-31061.

8. Gupta R, Ghosh S: **Phosphorylation of voltage-dependent anion channel by c-Jun N-terminal Kinase-3 leads to closure of the channel**. *Biochem Biophys Res Commun* 2015, **459**(1):100-106.

9. Abu-Hamad S, Arbel N, Calo D, Arzoine L, Israelson A, Keinan N, Ben-Romano R, Friedman O, Shoshan-Barmatz V: **The VDAC1 N-terminus is essential both for apoptosis and the protective effect of anti-apoptotic proteins**. *J Cell Sci* 2009, **122**(Pt 11):1906-1916.

10. Rasala BA, Orjalo AV, Shen Z, Briggs S, Forbes DJ: **ELYS is a dual nucleoporin/kinetochore protein required for nuclear pore assembly and proper cell division**. *Proc Natl Acad Sci U S A* 2006, **103**(47):17801-17806.

11. Laird AK: **Dynamics of tumor growth**. *Br J Cancer* 1964, **13(3)**:490-502.

12. Wang X, Niu J, Li J, Shen X, Shen S, Straubinger RM, Qu J: **Temporal effects of combined birinapant and paclitaxel on pancreatic cancer cells investigated via large-scale, ion-current-based quantitative proteomics (IonStar)**. *Mol Cell Proteomics* 2018, **17**(4):655-671.

13. Ernst J, Bar-Joseph Z: **STEM: a tool for the analysis of short time series gene expression data**. *BMC Bioinformatics* 2006, **7**:191.
